# Theoretical Investigations on Free Energy of Binding Cilostazol with Different Cyclodextrins as Complex for Selective PDE3 Inhibition

**DOI:** 10.3390/molecules29163824

**Published:** 2024-08-12

**Authors:** Marta Hoelm, Nilkanta Chowdhury, Sima Biswas, Angshuman Bagchi, Magdalena Małecka

**Affiliations:** 1 Department of Physical Chemistry, Faculty of Chemistry, University of Lodz, Pomorska 163/165, 90-236 Lodz, Poland; marta.hoelm@chemia.uni.lodz.pl; 2 Department of Biotechnology, Ranchi—Purulia Road Campus, Sidho-Kanho-Birsha University, Purulia 723104, West Bengal, India; nil5two@gmail.com; 3 Department of Biochemistry and Biophysics, University of Kalyani, Kalyani 741235, West Bengal, India; simabcbp18@klyuniv.ac.in

**Keywords:** cyclodextrins, cilostazol, cyclodextrin–cilostazol complex, ligand–protein interactions, molecular docking simulations, drug delivery, Gibbs free energy of solvation, DFT calculations

## Abstract

Cilostazol is a phosphodiesterase III inhibitor characterized by poor solubility. This limitation can be overcome by using a drug carrier capable of delivering the drug to the target site. Cyclodextrins are essential as drug carriers because of their outstanding complexation abilities and their capacity to improve drug bioavailability. This study comprises two stages: The first involves verifying different cyclodextrins and their complexation abilities towards cilostazol. This was accomplished using molecular docking simulations (MDS) and density functional theory (DFT). Both techniques indicate that the largest Sulfobutyl Ether-β-Cyclodextrin forms the most stable complex with cilostazol. Additionally, other important parameters of the complex are described, including binding sites, dominant interactions, and thermodynamic parameters such as complexation enthalpy, Gibbs free energy, and Gibbs free energy of solvation. The second stage involves a binding study between cilostazol and Phosphodiesterse3 (PDE3). This study was conducted using molecular docking simulations, and the most important energetic parameters are detailed. This is the first such report, and we believe that the results of our predictions will pave the way for future drug development efforts using cyclodextrin–cilostazol complexes as potential therapeutics.

## 1. Introduction

Cyclodextrins (CDs) are a group of oligosaccharides consisting of D-glucopyranose residues linked by α-1,4 glycosidic bonds, usually containing 6-12 D-glucopyranose units [1,2,3,4,5,6]. The three main cyclodextrins are α, β, and γ, which are composed of 6, 7, and 8 D-glucopyranose units, respectively (Figure S1 in the Electronic Supplementary Material (ESI)). Cyclodextrins have a toroidal shape with a hydrophobic cavity and a hydrophilic exterior part of different sizes [7] (Figure S1). The internal hydrophobic cavity leads to the formation of inclusion complexes with guest molecules through non-covalent interactions (van der Waals interaction or hydrogen bond) [8,9]. Hence, CDs are well known in supramolecular chemistry [10,11,12,13] similar to pseudorotaxanes [14,15,16,17,18]. Inclusion complexes of CDs can enhance the aqueous solubility [12,19,20,21], improve bioavailability [5,22], stability, and bioactivity of guest molecules and even reduce undesirable tastes [23,24,25]. For this reason, CDs find their uses in the food, pharmaceutical, material, agriculture, and chemical industries [26,27,28]. 

The inclusion processes in the cavities of CDs require the replacing of the solvent by a ligand molecule that is inserted into the cavity. Thus, this is a phenomenon of the combined effect of a dynamic improvement of conformational changes and intermolecular interactions [29,30]. Considering the above aspects, there is a great interest in understanding these phenomena through the characterizations of the inclusion complexes of CDs and thereby deciphering their properties at the molecular level. Consequently, many experimental techniques such as Ultraviolet Spectroscopy (UV), Nuclear Magnetic Resonance (NMR) [31], Infrared Spectroscopy (IR) [32], Raman spectroscopy [33,34,35], X-ray crystallography [36,37,38], and Isothermal Titration Calorimetry (ITC) [39,40,41,42] are used to characterize these inclusion systems. Computational analyses are being conducted side-by-side with experimental ones to supplement them. The molecular modeling approach, being an effective and popular method in supramolecular chemistry with cyclodextrin, comes as a suitable alternative to the highly expensive experimental techniques [43,44,45]. The origin of molecular docking comes from the lock and key concept [46]. However, the process of joining the ligand onto the active side of the receptor is much more complicated than the traditional lock–key model. Firstly, because of the continuous conformational changes in the ligand and the receptor, and secondly because of the compromise in the energy and spatial adjustment [47,48]. 

Cilostazol (CIL) is a vasodilatory drug with an antiplatelet effect. It is a Phosphodiesterase3 (PDE3) inhibitor [49], which inhibits the activity of phosphodiesterase in cyclic adenosine monophosphate (cAMP). As a result, it suppresses the degradation of cAMP and increases cAMP in platelets and blood vessels. These cAMPs can inhibit platelet vasodilation and aggregation. The absorption of cilostazol in the gastrointestinal tract is very slow and incomplete. Therefore, due to the fact that cilostazol has limited solubility, (~6 μg/mL at 25 °C) searching for new carriers for this drug is still ongoing [50]. Recently, cilostazol has also attracted attention due to its antiapoptotic, anti-inflammatory, antioxidant, and cardioprotective properties [51,52,53,54]. Multiple preclinical and clinical studies demonstrated that cilostazol could be used successfully in COVID-19 adjuvant therapy [55]. 

The aim of the present work was to obtain the information about intermolecular interactions of cilostazol with various derivatives of CDs as well as to investigate their most significant structural, energetic, and thermodynamic parameters. For this purpose, we used two different theoretical methods: the molecular docking simulations (MDS) and the density functional theory (DFT). So far, experimental data of the inclusion complex of cilostazol and β-CD were in hand, and our docking results were in good agreement with them. Gawali et al., [56] suggested the existence of 1:1 complex of CDs with the ligand cilostazol. Furthermore, there are experimental data for CDs in the literature where it was observed that CDs can act as potential carrier molecules [57,58]. Since substituted cyclodextrin derivatives such as HPβ-CD and SBEβ-CD are known to increase solubility of other drugs better than βCD, it is important to know the conditions of complex formation with cilostazol for the mentioned derivatives. As cilostazol is a PDE3 inhibitor, we tried to identify how cilostazol would interact with the PDE3. So far, this is the first such work where the binding interactions between the cilostazol and various CDs and PDE3 were analyzed. Our predictions aligned with the preliminary experimental data collected thus far. Therefore, our work may act as a baseline support to carry out bench works so that new drug delivery systems may come to light.

## 2. Results and Discussion

### 2.1. Docking and DFT of CDs:CIL 

**Figure 1:**
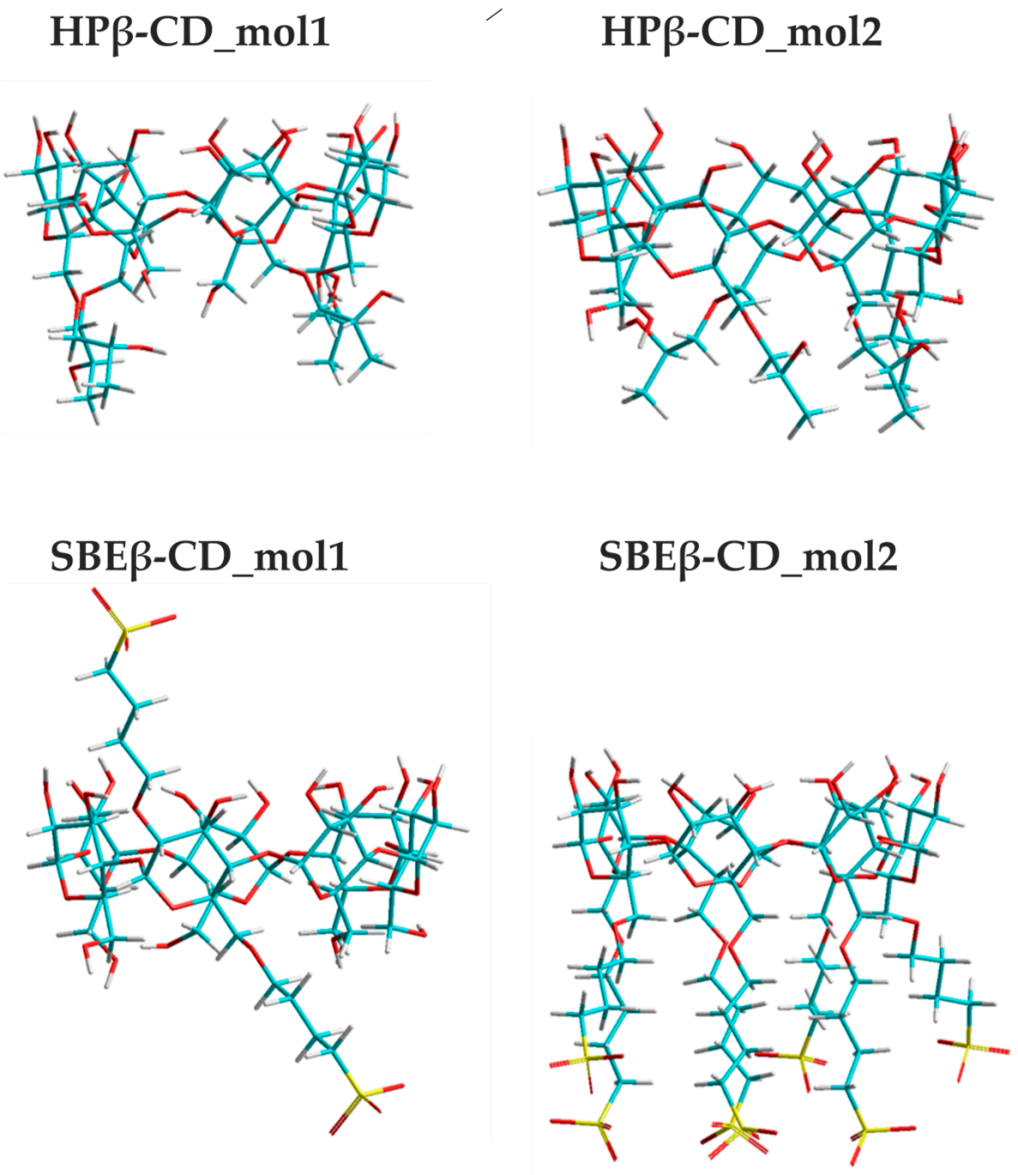
The molecules investigated in this study: Hydroxypropyl β-Cyclodextrin (mol1 and mol2) and Sulfobutyl Ether-β-Cyclodextrin (mol1 and mol2).

We used molecular docking simulations in order to study the binding interactions between α-CD, β-CD, HPβ-CD, and SBEβ-CD with the ligand cilostazol. The binding free energy values are presented in Table 1. However, for Hydroxypropyl β-Cyclodextrin and Sulfobutyl Ether-β-Cyclodextrin, we generated two different molecules, presented as molecule1 and molecule2 (Figure 1). Overall, the ligand cilostazol binds the best with SBEβ-CD_mol1, with a binding free energy of −6.85 kcal/mol. Between the different molecules of HPβ-CD, the molecule1 has slightly better binding interactions with cilostazol than molecule2. Both molecules of SBEβ-CD bind more strongly with the ligand cilostazol than HPβ-CD. 

It is to be noted that the Hydroxypropyl β-Cyclodextrin and Sulfobutyl Ether-β-Cyclodextrin had better binding interactions with the ligand cilostazol than the individual α- and β- Cyclodextrin. The presence of different substituents on the β-Cyclodextrin would make a conducive environment for the ligand cilostazol to interact in an a more favorable way. In the derivatives of β-CD, there are additional hydrophobic and hydrophilic moieties that might favor a better binding between them. For HPβ-CD, both molecules have nearly identical dispositions of the substituents. Therefore, they could interact with the ligand in a similar fashion, as exemplified by their almost equal binding free energy values. For SBEβ-CD, there are some alterations in the relative orientations of the substituents. Molecule1 has a wider cleft to fit in the ligand cilostazol as compared to molecule2. This might be a reason for cilostazol to have a better binding free energy with molecule1.

**Figure 2:**
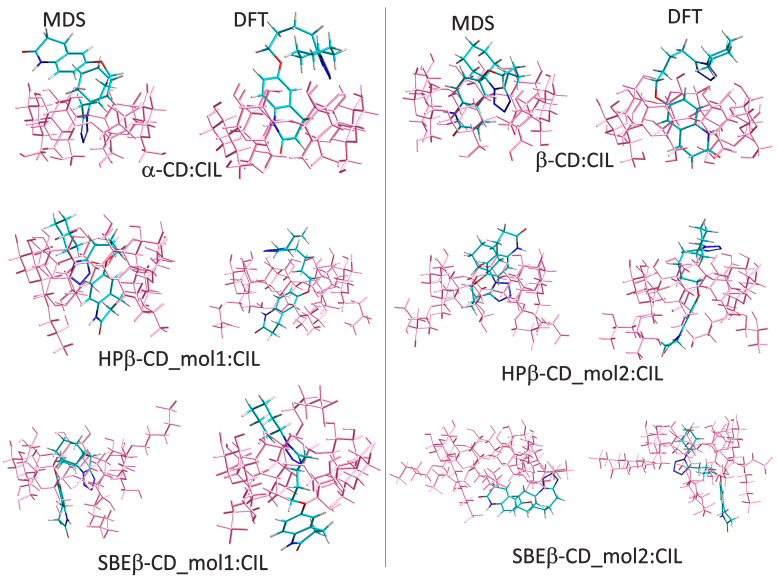
The most stable complexes of the CDs:CIL configurations obtained from the molecular docking simulations (MDS) and from the M06-2X-GD3/6-31G(d,p) calculations (DFT) performed in water. Atom colors: carbon—cyan, oxygen—red, nitrogen—dark blue, hydrogen—gray, cyclodextrins—pink. The coordinates of DFT structures are listed in Table S3 (ESI).

The results obtained from the quantum chemical calculations confirm the findings of the MDS study that the cyclodextrins form a very stable inclusion complex with cilostazol. This was rather expected, as CDs have excellent binding properties [59,60]. The most stable complexes of α-CD:CIL; β-CD:CIL; HPβ-CD_mol1:CIL; HPβ-CD_mol2:CIL; SBEβ-CD_mol1:CIL and SBEβ-CD_mol2:CIL were obtained from the M06-2X-GD3/6-31G(d,p) optimizations (DFT) and molecular docking simulations, as shown in Figure 2. It should be mentioned that during the configurational search (which includes AMBER99, PM7, and DFT calculations), three possible configurations of CIL inside the CD cavity (K01, K02, and K03) were taken into account. Their graphical representation is shown in Figure S2.

Although the complexes obtained from DFT and MDS differ in terms of geometry, a similarity is observed in the preferred position of CIL within the CD cavity. In the most energetically favorable configurations, the quinolinone ring (K01) or the butoxyl group (K02) of cilostazol is located inside the cavity of CDs. The exceptions are structures of SBEβ-CD_mol2:CIL where, according to DFT, the lowest energy is achieved when the cyclohexane ring (K03) is placed inside CD, which, compared to other moieties of CIL, has the largest size (see Figure 2). This can be attributed to the specific geometry of SBEβ-CD _mol2, which allows CIL to interact not only with the glucopyranose units, but also with the sulphobutyl chains (see Figure 2). This interaction is particularly evident in the case of the molecule obtained from MDS calculations. 

It should be mentioned that the DFT complexes presented in Figure 2 are characterized by the highest contribution to the population calculated from the Boltzmann analysis, with the values being listed in Table S1. In the latter, only the complexes with a contribution to the population of not less than 1% are presented. Additionally, it should be noted that all configurations (K01, K02, and K03) within each theoretical method were treated as a single population. As can be seen, the solution (M06-2X-GD3 calculations) may contain not only the most energetically preferable configurations but also other less stable ones. This trend is also reproduced by approximate methods such as AMBER99 and PM7 (see Table S1). The structures indicated by these methods as the most stable (with the highest contribution to the population) are shown in Figure S3. Also these methods indicate that the most energetically preferred configuration is the one in which the smaller moieties of CIL (quinolinone ring or the butoxyl group) are placed inside the CDs’ cavity. Again, the exception is SBEβ-CD, as in this case, the cyclohexane ring is located inside its cavity. 

**Figure 3:**
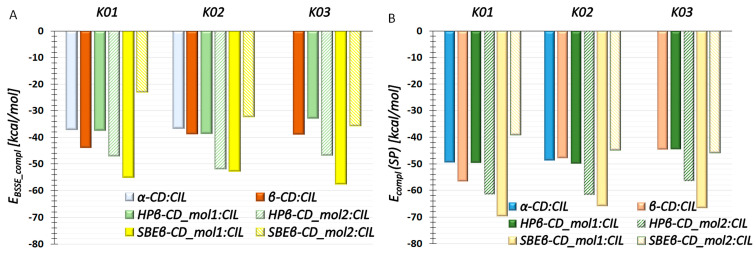
The BSSE corrected complexation energies (E_BSSE_compl_) obtained from the M06-2X-GD3/6-31G(d,p) optimizations (**A**) and from the single point calculations (E_compl_(SP); (**B**)) performed at the M06-2X-GD3/6-311G(d,p) theory level. The calculations were conducted in water (PCM) for the most energetically preferable structures selected from each configuration (K01; K02; K03;) of the various CDs:CIL complexes.

The stability of the complex is reflected in the values of the complexation energies, which are presented in Figure 3 for the structures shown in Figure 2. In general, the complexation energies were estimated for the structures optimized at the M06-2X-GD3/6-31G(d,p) theory level (E_BSSE_compl_), which contains the small basis set. However, our attempts to perform optimizations using a larger basis set (especially one containing diffusion functions) failed due to convergence issues with the system. Therefore, to verify the 6-31G(d,p) results, we performed single point (SP) calculations using the same functional but larger basis set 6-311G(d,p). SP calculations were conducted for the most stable optimized complexes selected from each (K01, K02, and K03) configuration.

According to results obtained from both calculations (OPT and SP), the smallest complexation energy is noted for the complexes of SBEβ-CD_mol1. It should be pointed out that this molecule was also indicated as the most energetically preferable by MDS. SBEβ-CD_mol2 forms less stable complexes than SBEβ-CD_mol1. The M06-2X-GD3 method suggests that the geometry of SBEβ-CD_mol2 promotes the formation of hydrogen bonds between the sulphobutyl chains. However, this HB network might be disrupted during the complexation process, leading to an increase in the energy of the cyclodextrin and, consequently, the entire complex. This could be the reason why, according to both methods DFT and MDS, this molecule forms less stable complexes (see Figure 3).

It should be emphasized that our study primarily focuses on the orientation of complexes where CIL is incorporated from the wider side of the CD cone. This is due to the fact that complexes with the drug placed from the narrower side of the cone are less energetically favorable [61]. Our recent work [62], which examines the complexes of loratadine with cyclodextrin, also confirms that such configurations exhibit higher complexation energies. However, to verify whether this trend also applies to cilostazol, we perform a test for one configuration of β-CD:CIL, namely K01* (Figure S4). For this complex, the BSSE-corrected complexation energy is −41.91 kcal/mol, so it is less stable by ~2 kcal/mol than β-CD:CIL_K01. Despite the small difference in stability, the observed trend was consistently replicated, probably because of the presence of the hydroxymethyl groups, which to some extent, block this entrance. 

In Table 2, for the most stable complexes selected from each configuration, the energetic and thermodynamic parameters obtained from the M06-2X-GD3/6-31G(d,p) optimizations and frequency calculations are listed. In turn, in Table S2, the corresponding values (without deformation), received from the single point calculations performed at the M06-2X-GD3/6-311G(d,p) theory level, are presented. It should be mentioned that the thermodynamic quantities presented in Table S2 ($H_{compl}^{SP}$ and $G_{corr\_compl}^{SP}$) were obtained by adding the M06-2X-GD3/6-31G(d,p) thermal corrections to the complexation energies E_compl_(SP) that were estimated from the single point calculations.

As can be seen in Table 2, the smallest value of E_defCD_ is noted for the cyclodextrin in the most stable complex SBEβ-CD_mol1_K03. This value is negative, indicating that the geometry of SBEβ-CD_mol1 in the complex, due to interactions with CIL, is more stable than its isolated structure. The formation of the HPβ-CD_mol2:CIL complex causes smaller distortion of the CD and CIL geometries than HPβ-CD_mol1:CIL, contributing to an increase in its stability. In the molecule, various types of interactions are present; however, the most visible are hydrogen bonds. They are mainly formed between OH groups of CD and the N or O atoms of cilostazol. The highest number is observed for the most stable complexes. For instance, HPβ-CD_mol2:CIL_K02 has six hydrogen bonds. Other less stable structures, such as α-CD complexes, have one or two HB. In Table 2, the complexation enthalpies $\left(H_{\mathrm{compl}}^{\mathrm{BSSE}}\right)$ and Gibbs energies ($G_{\mathrm{corr}\_\mathrm{compl}}^{\mathrm{BSSE}}$) are also presented, with negative values indicating that the complexation process in all cases is exothermic and spontaneous, respectively.

The spontaneous process of complex formation is also confirmed by the values of chemical potential (μ), which, along with other parameters of complex reactivity (hardness (η) and global electrophilicity index (ω)), are presented in Table S4. The electronic chemical potential also provides information about the charge-transfer process that occurs upon complex formation [63]. Electrons flow from a molecule of higher μ to a molecule of lower μ. Therefore, electrons will be transferred from cyclodextrin to the complex. Chemical hardness and the HOMO-LUMO gap inform the chemical reactivity [64], and as can be seen in Table S4, the most reactive cyclodextrin is SBEβ-CD_mol2. Interestingly, α-CD, β-CD, and HPβ–CD_mol2 exhibit nearly identical reactivity, as their values are within the same order of magnitude. 

**Figure 4:**
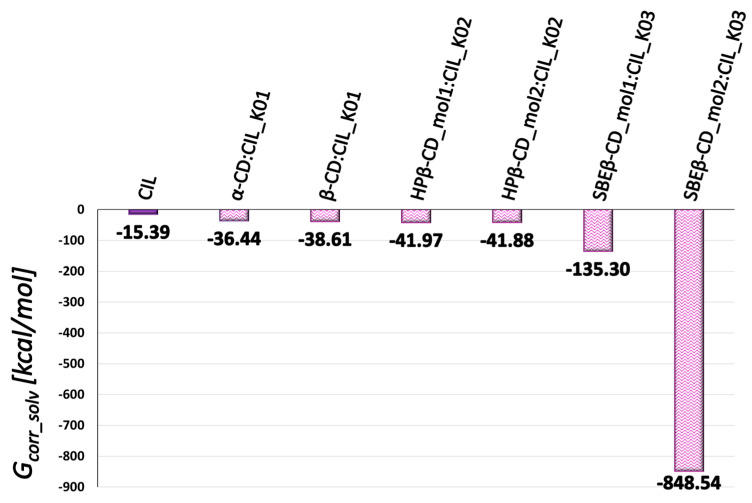
The values of the Gibbs energy of solvation (G_corr_solv_) obtained for the cilostazol (CIL) and the most stable complexes from each configuration.

One of the most important thermodynamic parameters characterizing a molecule in a solution is solvation. It is the process wherein solvent molecules attract and combine with the molecules or ions of the solute. When this process occurs in water, it is termed hydration [65]. From a theoretical point of view, the effect of solvation can be evaluated by calculating the Gibbs energy of solvation (G_corr_solv_) [66]. The latter is shown in Figure 4 for cilostazol and the most stable complexes selected from each configuration and in Table S5 for isolated cyclodextrins. 

Cilostazol is poorly soluble in water [50], so it is not surprising that its absolute G_corr_solv_ value is small. The complexes are significantly much better solvated; however, it should be emphasized that in this aspect, SBEβ-CD_mol2 takes the lead. It also exhibits the highest absolute value of the hydration Gibbs solvation (Table S5), which was rather expected since, generally, this cyclodextrin is significantly more soluble in water than β-CD [67].

### 2.2. MD Simulations of SBEβ-CD_mol1:CIL

**Figure 5:**
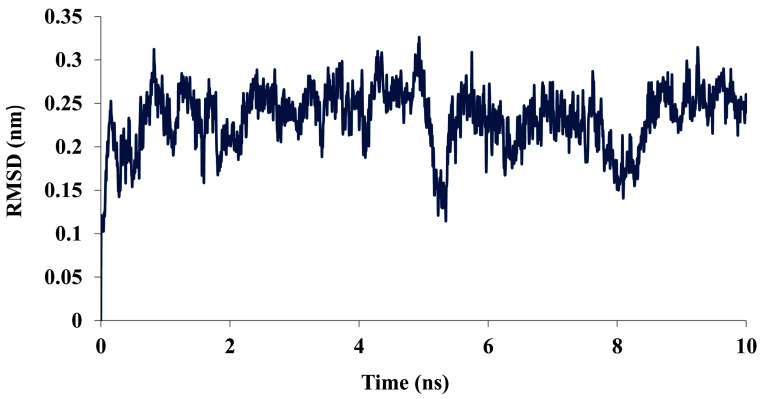
All-atom RMSD plot of SBEβ-CD_mol1:CIL complex.

In order for the CD:CIL complexes to work in aqueous environments, they should be able to withstand the surrounding pressure. We performed a MD simulation of the SBEβ-CD_mol1:CIL, as this complex has the strongest binding interactions among all the CD:CIL complexes. The progress of the MD simulation run was monitored by plotting the all-atom RMSD of the ligand complex with time (Figure 5).

### 2.3. Docking of CIL:PDE3

**Figure 6:**
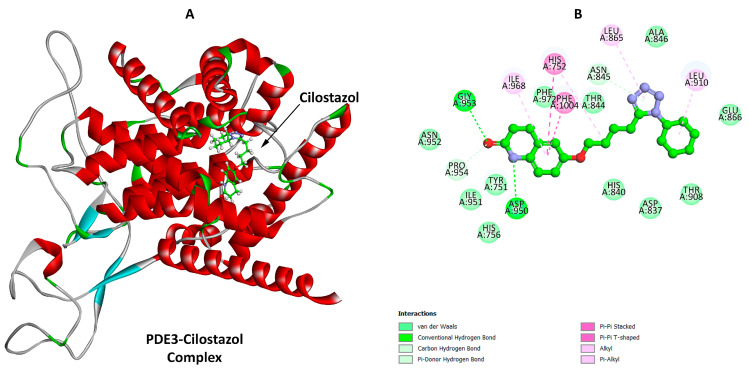
The binding interactions between PDE3 and cilostazol. The left panel (**A**) shows the protein PDE3 in cartoon and the cilostazol in ball and stick. The right panel (**B**) shows the amino acid residues of PDE3 undergoing different types of non-covalent interactions with the cilostazol.

We computed the binding interactions between Phosphodiesterase3 (PDE3) in a monomeric condition with the ligand cilostazol. The binding free energy (ΔG) of the interaction between cilostazol and PDE3 monomer was found to be −8.79 kcal/mol as obtained from Autodock 4.2 [68]. The different types of binding interactions present between the ligand and PDE3 are depicted in Figure 6. It can be seen from our study that the cilostazol binds to the ligand binding pocket of PDE3 (Figure 6).

### 2.4. MD Simulations of CIL:PDE3

**Figure 7:**
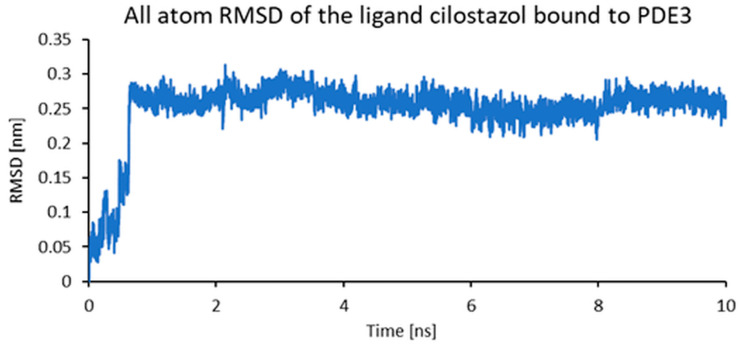
The plot of RMSD of all atoms of cilostazol with time when bound to PDE3. The ligand was found to become stabilized after 3 ns.

In order to check the dynamicity of the ligand cilostazol when bound to PDE3, we performed a molecular dynamic simulation of the PDE3-Cilostazol complex. The pro-gress of the dynamics run was monitored by plotting the deviations of the atoms (all atoms present in the ligand) of cilostazol with time. We observed that the ligand, when bound to PDE3, would start attaining stability after 3 ns of the simulation run (Figure 7).

As observed from Figure 5, the ligand complex has not yet stabilized until the end of the simulation run, 10 ns. On the other hand, Figure 7 clearly depicts that the ligand cilostazol would remain stably bound to PDE3, and it started attaining its stability after 3 ns of the MD simulation run. Therefore, it may be safely concluded that the ligand cilostazol interacts in a better way with PDE3 than with Sulfobutyl Ether-β-Cyclodextrin_mol1. This might be due to the fact that cilostazol would remain enclosed within PDE3, where there is a conducive distribution of polar and non-polar amino acid residues. This could point towards the fact that the derivative of CD, Sulfobutyl Ether-β-Cyclodextrin_mol1), is not able to bind to cilostazol as strongly as PDE3. Furthermore, in the presence of PDE3, the cilostazol may come out of the CD:CIL complex to bind the protein. Thus, the different derivatives of CD may serve the purpose as a carrier of cilostazol.

## 3. Materials and Methods

### 3.1. Docking and Quantum Chemical Calculations of CDs:CIL

For docking simulation studies, the coordinates of cilostazol, α-CD and β-CD were obtained from the Crystallographic Cambridge Structural Database [69]. The appropriate refcodes are as follows: α-CD (refcode: CHXAMH02 [70]); β-CD (refcode: BCDEXD05 [71]); CIL (refcode: XOSGUH01 [72]). α-CD and β-CD contain water molecules, which were removed. Their geometrical representations are shown in Figures S5 and S6 for cyclodextrins and CIL, respectively. HPβ-CD and SBEβ-CD were prepared in the HyperChem [73] program by adding hydroxypropyl groups (4 groups) or sulfobutyl groups (2 and 7 groups for the SBEβ-CD_mol1 and SBEβ-CD_mol2 molecules, respectively) at the wider edge of the glucopyranose unit belonging to β-CD. Their geometrical representations are shown in Figure 1. All the geometries of the compounds were further optimized in Discovery Studio (DS) [74] before performing the docking simulation.

We used AutoDock 4 [68] and performed blind docking between cilostazol and CDs, using Lamarckian Genetic Algorithm (GA) and 100 GA runs for each docking simulation. The box size and the grid center were used as follows:

α-CD:CIL: Box size (40, 40, 40) Å^3^ and grid center (−3.696, 3.94, 2.929),

β-CD:CIL: Box size (40, 40, 40) Å^3^ and grid center (−0.961, 0.705, 0.003),

HPβ-CD_mol1:CIL: Box size (50, 50, 50) Å^3^ and grid center (22.463, 14.268, 11.376),

SBEβ-CD_mol1:CIL: Box size (50, 50, 50) Å^3^ and grid center (16.173, 12.613, 12.574).

In these cases, we considered molecule1 of both Hydroxypropyl β-Cyclodextrin and Sulfobutyl Ether-β-Cyclodextrin.

Then, we performed a second round of docking simulations, where we considered the second molecule of Hydroxypropyl β-Cyclodextrin and Sulfobutyl Ether-β-Cyclodextrin. We followed the same protocol for docking as mentioned above. However, the box sizes and coordinates of the grid centers are as follows:

HPβ-CD_mol2:CIL: Box size (50, 50, 50) Å^3^ and grid center (22.463, 20.004, 11.376),

SBEβ-CD_mol2:CIL: Box size (60, 50, 70) Å^3^ and grid center (20.632, 16.544, 21.754).

**Chart 1:**
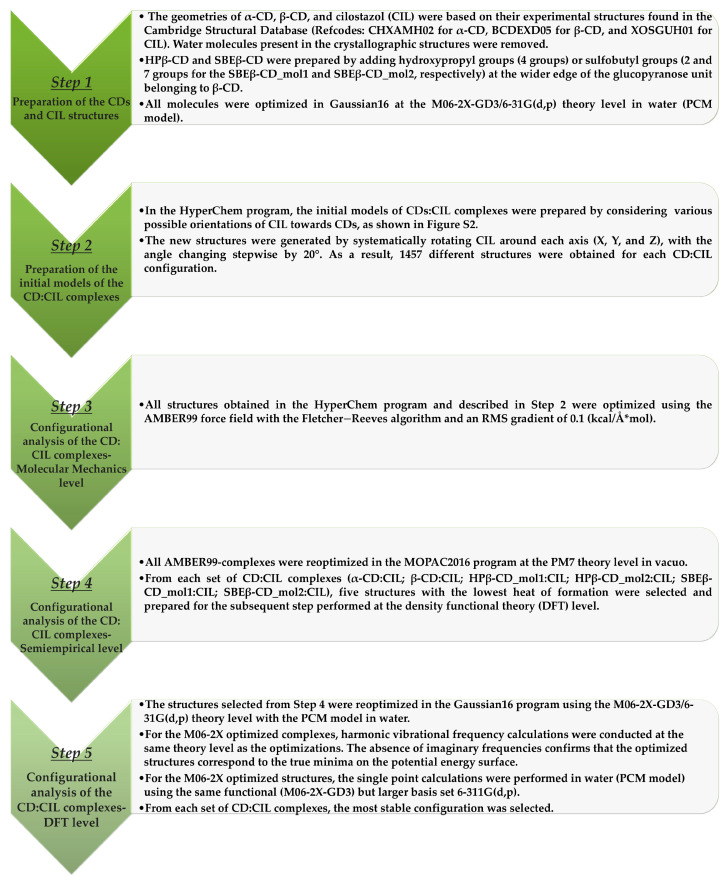
Flow chart describing the computational strategy applied in the configurational search performed to obtain the most stable complexes of CDs:CIL.

The quantum chemical calculations were conducted to obtain a more reliable description of the most important structural and energetical properties of the various CDs complexes with cilostazol. However, to achieve this, it is first necessary to find the most stable configuration of each complex. The search for the most energetically preferable structure was carried out by means of a configurational analysis, which was divided into three steps in our study. Each step is characterized by a different yet increasing level of theory used in the calculations. This procedure is schematically presented in Chart 1.

The first is associated with the generation of CDs complexes and the molecular mechanics calculations. The initial models of the CDs:cilostazol complexes were built in the HyperChem program, using the coordinates of CDs and cilostazol as above. In the next step, their geometries were optimized at the M06-2X-GD3/6-31G(d,p) theory level in water (PCM model). Details of this methodology are outlined in the final step of the configuration analysis provided below. In the initial configuration of complexes, various possible orientations of CIL towards CDs were taken into account, as shown in Figure S2. Note that, for only one configuration of the β-CD complex (K01), we consider two orientations in which CIL approaches cyclodextrin from both entrances (wider and narrower). This is due to the fact that introducing the drug into the CD cavity from its narrower side is much less energetically favorable [62,75,76]. The new structures were produced by a systematic rotation of CIL around each axis, X, Y, and Z, and changing the angle stepwise by 20°. Consequently, 1457 different structures for each CDs:CIL configuration were obtained and optimized in the HyperChem program using the AMBER99 [77] force field. The latter is designed to study the biological macromolecules, including sugar derivatives. Although molecular mechanics is the approximate method that does not treat electrons explicitly, it allows for detailed exploration of the configurational surface. 

In the second step, all complexes obtained from the AMBER99 calculations were reoptimized in the MOPAC2016 program [78] at the PM7 [79] theory level in vacuo. The PM7-optimized structures were ranked according to the increasing heats of formation (ΔH_f_). Five structures from each configuration of all CDs:CIL complexes with the lowest value of ΔH_f_ were selected, giving a total of 95 different complexes. These structures were, in the next step, reoptimized at the density functional theory (DFT) level using the meta exchange-correlation functional (M06-2X) [80] with the addition of Grimme’s empirical pairwise long-range (dispersion) corrections (GD3) [81] and Pople’s basis set 6-31G(d,p) [82]. The choice of the DFT method was justified by its good performance in predicting the structural and energetical parameters of various cyclodextrin systems [83,84,85,86,87]. The M06-2X-GD3/6-31G(d,p) calculations were performed in the presence of water described by the polarizable continuum model of solvent PCM. The harmonic vibrational frequency calculations were performed at the same theory level to confirm that the optimized structures correspond to an energy minimum at the potential energy surface and to obtain the values of enthalpy (H) and Gibbs energy (G). The later were recalculated in the GoodVibes program using the Grimme approach [88] to include corrections to the entropy term for low vibrational frequencies <100 cm^−1^. The corrected values of Gibbs energies are denoted as G_corr_.

The stability of the complexes and the strength of interactions were assessed by calculating the complexation (E_compl_) and interaction (E_int_) energies using the supermolecular approach as follows:

(1) $$E_{compl}=E_{complex}^{OPT}-\left(E_{CD}^{OPT}+E_{CIL}^{OPT}\right)$$

(2) $$E_{int}=E_{complex}^{OPT}-(E_{CD}^{SP}+E_{CIL}^{SP})$$

where $E_{\mathrm{complex}}^{\mathrm{OPT}}$ is the energy of the optimized CDs:CIL complex; $E_{\mathrm{CIL}}^{\mathrm{OPT}}\mathrm{and}E_{\mathrm{CD}}^{\mathrm{OPT}}$ are the energies of the optimized structures of CIL and various CDs, respectively; $E_{\mathrm{CD}}^{\mathrm{SP}}\;\mathrm{and}\;E_{\mathrm{CIL}}^{\mathrm{SP}}$ are the single point energies of CDs and CIL, respectively, extracted from the optimized complex obtained from the PCM calculations. The energy parameters were additionally corrected for the basis set superposition error (BSSE) using the full counterpoise method [89]. 

For the most stable complexes, as well as for the isolated cyclodextrins and CIL, obtained from the M06-2X-GD3/6-31G(d,p) optimizations, the single point (SP) calculations were performed at the M06-2X-GD3/6-311G(d,p) theory level in water (PCM). From the SP calculations, the complexation energies E_compl_(SP) were estimated. The thermodynamic quantities ($H_{compl}^{SP}$ and $G_{corr\_compl}^{SP}$) were calculated by adding the M06-2X-GD3/6-31G(d,p) thermal corrections (obtained from the frequency calculations) to E_compl_(SP) values.

The reactivity indicators, such as chemical potential (μ), hardness (η), and global electrophilicity index (ω) were calculated according to the following equations:

(3) $$\mu =\frac{E_{\mathrm{HOMO}}+E_{\mathrm{LUMO}}}{2}$$

(4) $$\eta =\frac{E_{\mathrm{LUMO}}-E_{\mathrm{HOMO}}}{2}$$

(5) $$\omega =\frac{\mu^{2}}{2\eta }$$

where E_HOMO_ and E_LUMO_ indicate the values of the highest occupied molecular orbital (HOMO) and the lowest unoccupied molecular orbital (LUMO), respectively, obtained from the M06-2X-GD3/6-31G(d,p) calculations.

The Gibbs energy of solvation G_corr_solv_ was determined as follows: G_corr_solv_ = G_corr_WAT_ − G_corr_GP_ where G_corr_WAT_ and G_corr_GP_ represent the values of Gibbs energy obtained during the M06-2X-GD3/6-31G(d,p) calculations performed in water (PCM) and in gas phase, respectively. All DFT calculations were performed in the Gaussian16 program [90].

### 3.2. Molecular Dynamics Simulation of SBEβ-CD_mol1:CIL Complex 

We performed the MD simulation of the SBEβ-CD_mol1:CIL complex in order to study its dynamicity in a solution. We chose this complex as this one showed the best binding interactions between all the CD:CIL complexes. In the first step, we used SwissParam [91] for the generation of the topologies of the ligands with the help of MMFF [92]. For MD simulations, we used the GROMACS program [93]. The entire system was placed in a periodic cubic box with the dimensions of (50 × 50 × 50) Å^3^ and dissolved in a SPC216 water model and thereby followed a similar protocol as mentioned below.

### 3.3. Docking of CIL:PDE3

We used the 3-D Phosphodiesterase3 molecule from PDB, bearing the PDB ID: 7LRC, with a resolution of 2.97 Å as the receptor for the ligand cilostazol. We filled in the missing residues in 7LRC via molecular modeling with the help of SwissModel [94] and refined the structure by energy minimization using DS. The amino acid sequence comparison between the built model (Model_PDE3) and the crystal structure 7LRC (PDE_7LRC) is presented in Supplementary Figure S7. We checked the stereochemical fitness of the structure and then used the refined structure for docking simulations.

Docking simulations were performed in Autodock 4, using Lamarckian Genetic Algorithm (GA) and 100 GA runs for each docking simulation. We did the docking with the monomeric structure of PDE3 with the ligand cilostazol. The grid box size was (200 × 160 × 240) Å^3^, and the coordinates of the grid center were (157.017, 159.705, 196.687). 

### 3.4. MD of CIL:PDE3

After that, the docked complex having the best score from Autodock 4 was considered for MD simulation. We used the GROMACS program for this purpose. The ligand topology was generated using SwissParam. We used a CHARMM27 [95] force field to simulate the PDE3:CIL complex. To solvate the system, we used the SPC (E) water model. The entire system of the PDE3:CIL complex was placed in a periodic cubic box with the dimensions of (106.04 × 106.04 × 106.04) Å^3^. Then, the system was energy-minimized. We then equilibrated the system performing NPT and NVT simulations for 100 picoseconds (ps). The system, when it became fully relaxed, was subjected to a MD production run for 10 nanoseconds (ns). The all-atom RMSD of the ligand cilostazol was plotted with time. 

## 4. Conclusions

In this study, we successfully docked the cilostazol ligand to α-Cyclodextrin (α-CD), β-Cyclodextrin (β-CD) and its modified derivatives, the Hydroxypropyl β-Cyclodextrin (HPβ-CD) and Sulfobutyl Ether-β-Cyclodextrin (SBEβ-CD) systems. The most significant properties of these complexes were studied using two different theoretical methods: density functional theory (DFT) and molecular docking simulations (MDS). Although all cyclodextrins form very stable complexes, cilostazol is most strongly bound by SBEβ-CD and HPβ-CD. These structures possess additional chains: Sulphobutyl (SBEβ-CD) or Hydroxypropyl (HPβ-CD), which are also involved in the interactions with CIL, positively influencing the stability of the complex. As indicated by the M06-2X-GD3 method, the most energetically preferred orientation is when the quinolinone ring or butoxyl group of cilostazol are incorporated into the cavity of CDs from the wider side of the cone. For all investigated cases, the values of the complexation enthalpies $\left(H_{\mathrm{compl}}^{\mathrm{BSSE}}\right)$ and Gibbs free energies ($G_{\mathrm{corr}\_\mathrm{compl}}^{\mathrm{BSSE}})$ are found to be negative, suggesting that the formation of the CDs:CIL complex is exothermic and spontaneous. Moreover, the calculated Gibbs free energy of the solvation G_corr_solv_ indicates that cilostazol is characterized by a very small absolute value, which is increased when CIL is bound to SBEβ-CD in the complex.

To analyze the mode of interactions of cilostazol with PDE3, we docked the cilostazol molecule as the ligand to the receptor PDE3. PDE3 is a homodimer. So, we first docked the ligand with one of the monomers and then with the entire dimer. In both cases, cilostazol becomes attached to the ligand-binding pocket of PDE3. As observed from the docking simulations, the ligand cilostazol has its affinity only towards the ligand-binding pocket of PDE3. This would strengthen our belief that in the presence of a good carrier like cyclodextrins or its derivatives, the ligand cilostazol may reach its proper zone of interactions in PDE3 and would bind to the ligand-binding pocket. This might offset the possible off target interactions of the ligand. Furthermore, the MDS study of D_SBEβ-CD_mol1:CIL complex could point towards the fact that cilostazol would remain stably bound to PDE3 as compared to the Sulfobutyl Ether-β-Cyclodextrin_mol1. Therefore, it may be safely concluded that the ligand cilostazol first binds to Sulfobutyl Ether-β-Cyclodextrin_mol1 and is then carried to PDE3, where it is released to attach itself to PDE3. 

So far, this is the first report on the binding interactions between CDs with cilostazol as well as with PDE3. From our analysis, it could be predicted that HPβCD and SBEβ-CD may serve as effective encapsulating agents for CIL, particularly in the putative molecules 1 and 2, as presented in Figure 3. The encapsulated cilostazol may easily become dissolved in the body fluids and can exert its potential inhibitory activity against PDE3. Therefore, the problem of low solubility of cilostazol can be circumvented by obtaining it in either in HPβ-CD_mol1 or SBEβ-CD_mol1. Therefore, our study may be used for future drug designing endeavors. 

## Figures and Tables

**Table 1:** Binding interactions between CDs with the ligand cilostazol.

CDs:CIL	Binding Free Energy ΔG [kcal/mol]
α-CD:CIL	−4.24
β-CD:CIL	−5.49
HPβ-CD_mol1:CIL	−5.97
SBEβ-CD_mol1:CIL	−6.85
HPβ-CD_mol2:CIL	−5.92
SBEβ-CD_mol2:CIL	−6.08

**Table 2:** The BSSE corrected interaction energies ($E_{int}^{BSSE}$), complexation enthalpies ($H_{compl}^{BSSE}$), and complexation Gibbs energies ($G_{corr\_compl}^{BSSE}$) as well as the deformation energies (*E_def_*) for the most stable CDs:CIL complexes obtained in water (PCM) from the M06-2X-GD3/6-31G(d,p) optimizations. All values are in kcal/mol.

Parameter	α-CD:CIL_K01	β-CD:CIL_K01	HPβ-CD_mol1:CIL_K02	HPβ-CD_mol2:CIL_K02	SBEβ-CD_mol1:CIL_K03	SBEβ-CD_mol2:CIL_K03
$E_{int}^{BSSE}$	−44.84	−62.93	−56.61	−61.41	−56.79	−51.45
*E_defCD_*	7.09	17.36	11.93	5.61	−4.80	11.35
*E_defCIL_*	0.62	1.74	6.06	4.01	4.03	4.53
*E_def_tot_*	7.71	19.10	18.00	9.62	−0.78	15.88
$H_{compl}^{BSSE}$	−34.44	−40.07	−36.03	−49.40	−54.05	−33.71
$G_{corr\_compl}^{BSSE}$	−20.52	−22.66	−19.72	−32.30	−34.40	−20.71

## Data Availability

The original contributions presented in the study are included in the article and Supplementary Material, further inquiries can be directed to the corresponding authors.

## Supplementary Materials

The following supporting information can be downloaded at https://www.mdpi.com/article/10.3390/molecules29163824/s1, Figures S1–S7; Tables S1–S5. The structure of α-CD, β-CD, γ-CD, The initial configurations of the CDs:CIL, The most energetically privileged structures of each CDs:CIL configuration obtained during the configurational search performed at AMBER99 and PM7, β-CD:CIL_K01* optimized in water (PCM), The initial configurations of the CDs and CIL, Amino acid sequence comparison, The relative contributions to the population obtained from the Boltzmann distribution, The interaction and complexation energies, The cartesian coordinates, HOMO LUMO energies, The Gibbs energy.
