# Supplementary material for: Theoretical Investigations on Free Energy of Binding Cilostazol with Different Cyclodextrins as Complex for Selective PDE3 Inhibition
Source: Molecules. 2024 Aug 12;29(16):3824. doi: 10.3390/molecules29163824 (PMC11357564; doi:10.3390/molecules29163824)
Supplement: Supplementary file 1 [file molecules-29-03824-s001.zip › molecules-3100968-supplementary.pdf]

Supplementary material

Theoretical Investigations on Free Energy of Binding Cilostazol with Different Cyclodextrins as Complex for Selective PDE3 Inhibition

Marta Hoelm [1], Nilkanta Chowdhury [2], Sima Biswas [3], Angshuman Bagchi [3,*] and Magdalena Małecka [1,*]

[1] Department of Physical Chemistry, Faculty of Chemistry, University of Lodz, Pomorska 163/165, 90-236 Lodz, Poland; marta.hoelm@chemia.uni.lodz.pl
[2] Department of Biotechnology, Sidho-Kanho-Birsha University, Ranchi—Purulia Road Campus, Purulia 723104, West Bengal, India; nil5two@gmail.com
[3] Department of Biochemistry and Biophysics, University of Kalyani, Kalyani 741235, West Bengal, India; simabcbp18@klyuniv.ac.in
* Correspondence: angshu@klyuniv.ac.in (A.B.); magdalena.malecka@chemia.uni.lodz.pl (M.M.)

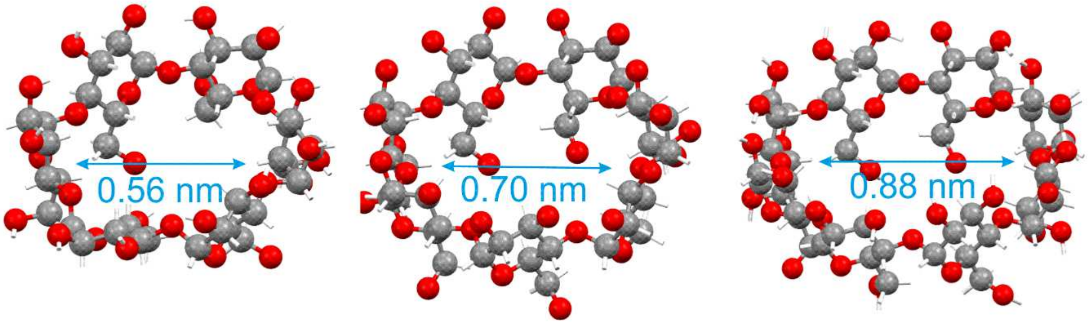

Figure S1. Schematic chemical structures of the three main types of cyclodextrins.

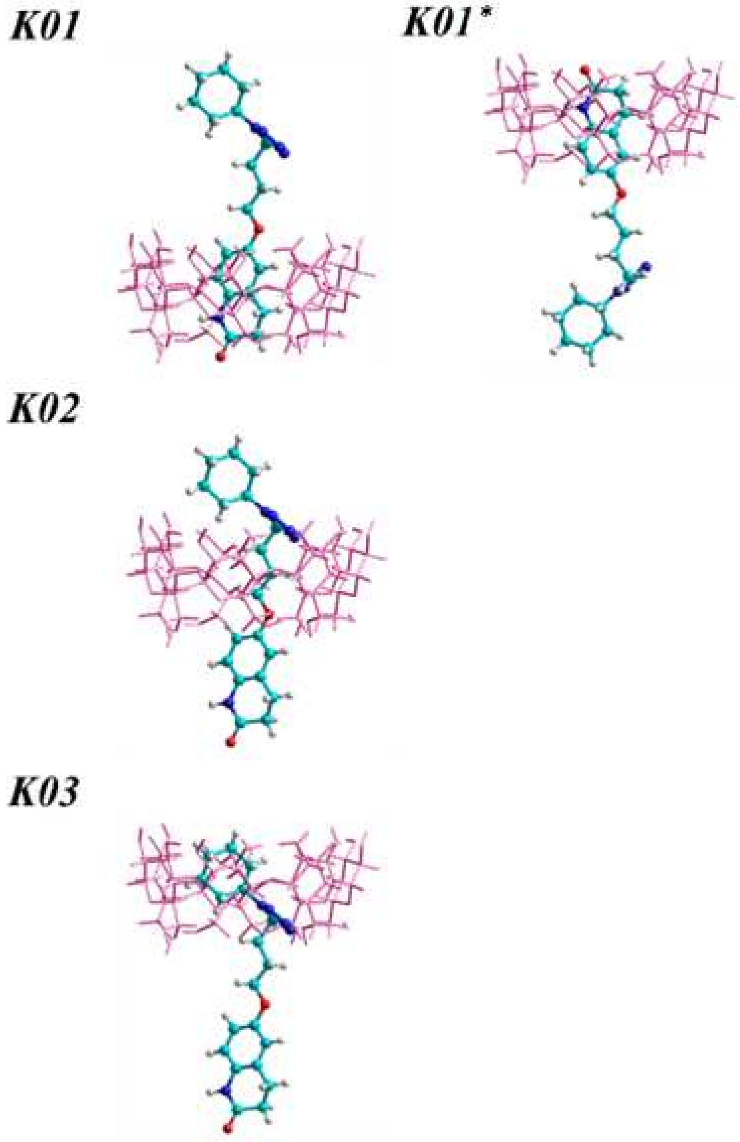

Figure S2. The initial configurations of the CDs:CIL complexes considered during the first step of the configurational search. Cyclodextrin is highlighted in pink.

Table S1. The relative contributions to the population obtained from the Boltzmann distribution in terms of energy differences (ΔE) for complexes received from the AMBER99, PM7 and M06-2X-GD3/6-31G(d,p) calculations.

**Complex**	**ΔE [kcal/mol]**	**contribution [%]**
	**AMBER99**	
α-CD:CIL_1	0.000	88
α-CD:CIL_2	1.447	8
α-CD:CIL_3	2.135	2
β-CD:CIL_1	0.000	70

β-CD:CIL_2	1.004	13
HPβ-CD_mol1:CIL_1	0.000	87
HPβ-CD_mol1:CIL_2	2.258	2
HPβ-CD_mol2:CIL_1	0.000	73
HPβ-CD_mol2:CIL_2	1.223	9
HPβ-CD_mol2:CIL_3	2.012	2
SBEβ-CD_mol1:CIL_1	0.000	81
SBEβ-CD_mol1:CIL_2	1.005	15
SBEβ-CD_mol1:CIL_3	2.053	3
SBEβ-CD_mol2:CIL_1	0.000	72
SBEβ-CD_mol2:CIL_2	0.950	15
SBEβ-CD_mol2:CIL_3	1.519	6
	**PM7**	
α-CD:CIL_1	0.000	96
α-CD:CIL_2	1.912	4
β-CD:CIL_1	0.000	96
β-CD:CIL_2	2.097	3
HPβ-CD_mol1:CIL_1	0.000	99
HPβ-CD_mol2:CIL_1	0.000	97
HPβ-CD_mol2:CIL_2	2.226	2
SBEβ-CD_mol1:CIL_1	0.000	98
SBEβ-CD_mol1:CIL_2	2.506	1
SBEβ-CD_mol2:CIL_1	0.000	98
SBEβ-CD_mol2:CIL_2	2.383	2
	M06-2X-GD3/6-31G(d,p)	
α-CD:CIL_1	0.000	86
α-CD:CIL_2	1.248	10
α-CD:CIL_3	2.031	3
β-CD:CIL_1	0.000	72
β-CD:CIL_2	0.561	28
HPβ-CD_mol1:CIL_1	0.000	85
HPβ-CD_mol1:CIL_2	1.061	14
HPβ-CD_mol2:CIL_1	0.000	83

HPβ-CD_mol2:CIL_2	0.949	17
SBEβ-CD_mol1:CIL_1	0.000	78
SBEβ-CD_mol1:CIL_1	0.772	21
SBEβ-CD_mol2:CIL_1	0.000	99

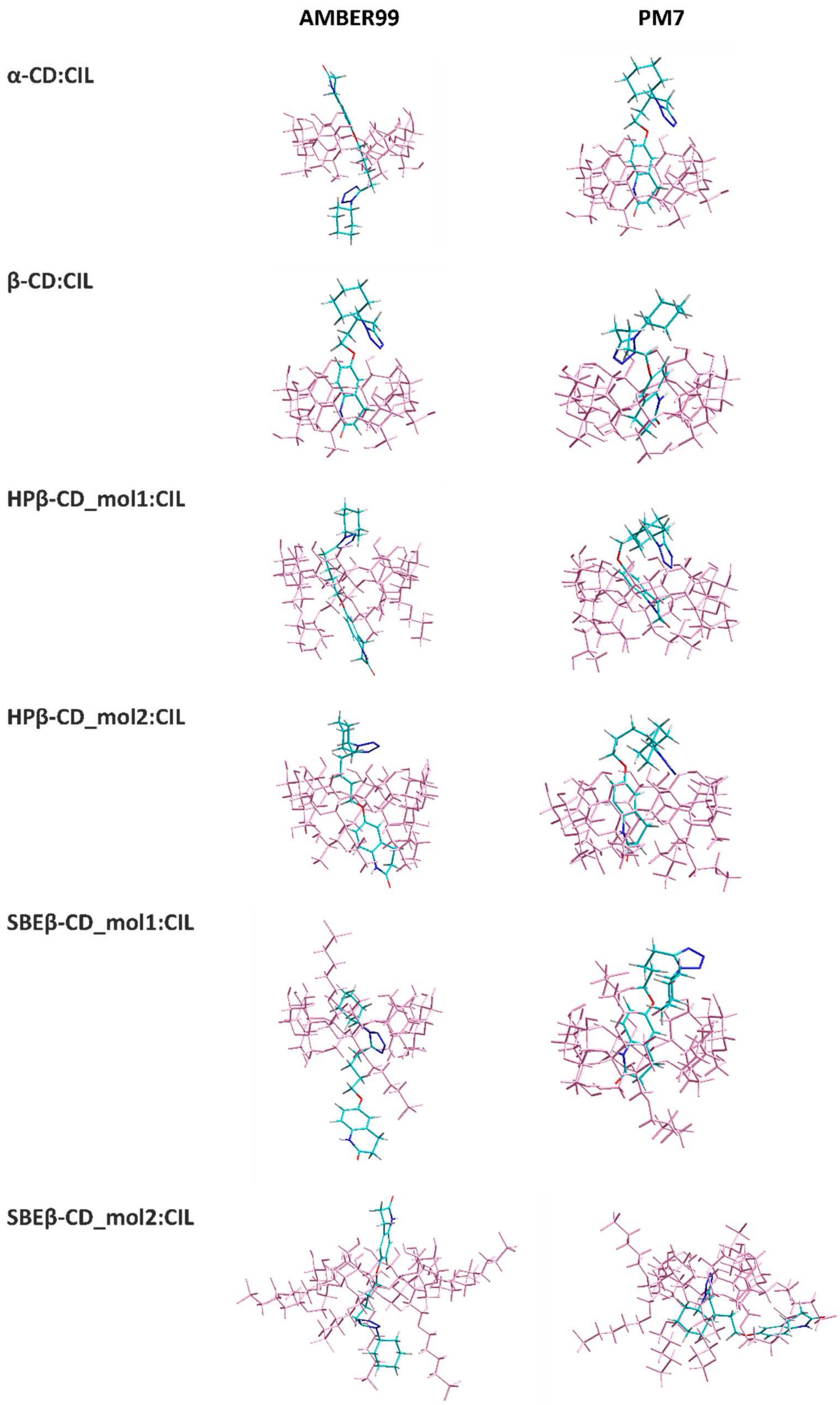

Figure S3. The most energetically privileged structures of each CDs:CIL configuration obtained during the configurational search performed at AMBER99 and PM7 theory levels in vacuo.

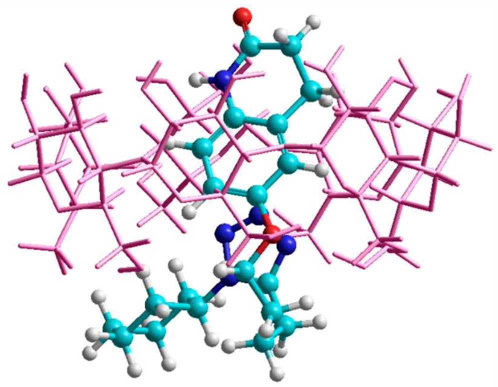

Figure S4. The β-CD:CIL_K01* complex structure obtained from the M06-2X-GD3/6-31G(d,p) optimizations in water (PCM).

Table S2. The interaction energies (E_{int}^{SP}) estimated from the single point calculations performed at the M06-2X-GD3/6-311G(d,p) theory level for the structures obtained from the M06-2X-GD3/6-31G(d,p) optimizations. The thermodynamic quantities (H_{compl}^{SP} and $G_{corr\_compl}^{SP}$) were obtained by adding the M06-2X-GD3/6-31G(d,p) thermal corrections to the complexation energies E_{compl}(SP) estimated from the single point calculations. All values are in kcal/mol.

Parameter	α-CD:CIL_K01	β-CD:CIL_K01	HPβ-CD_mol1:CIL_K02	HPβ-CD_mol2:CIL_K02	SBEβ-CD_mol1:CIL_K03	SBEβ-CD_mol2:CIL_K03
E_{int}^{SP}	-56.6	-76.0	-68.5	-71.8	-65.8	-62.2
H_{compl}^{SP}	-46.6	-52.7	-47.0	-58.9	-62.3	-43.5
$G_{corr\_compl}^{SP}$	-32.8	-36.7	-31.4	-42.8	-44.0	-30.5

Table S3. Cartesian coordinates [Å] of the most stable inclusion CDs:CIL complexes obtained from the M06-2X-GD3/6-31G(d,p) optimizations in water (PCM) and presented in Figure 2 in the main article.

	α-CD:CIL_K01				*β-CD:CIL_K01*		
**Atom**	**x [Å]**	**y [Å]**	**z [Å]**	**Atom**	**x [Å]**	**y [Å]**	**z [Å]**
C	2.83871	-3.11666	-2.91477	C	4.37017	3.51088	-2.21721
C	4.12768	-2.90933	-2.11982	C	4.47405	4.62667	-1.17935
C	4.25382	-1.45420	-1.71022	O	5.57315	4.37461	-0.31768
C	4.05946	-0.47531	-2.86488	C	3.19455	4.68769	-0.36965
C	2.81903	-0.86393	-3.66769	O	3.32056	5.76118	0.54101
C	2.63898	-0.12124	-4.97471	C	1.98326	4.82743	-1.28297
C	-1.98586	-4.51725	-1.28397	O	0.80846	4.55100	-0.53777
C	-0.77439	-5.19332	-0.63380	C	2.01891	3.79234	-2.40650

C	0.44261	-4.27645	-0.67350	O	3.28945	3.77888	-3.06361
C	0.64877	-3.76295	-2.09303	C	0.98721	4.06899	-3.48162
C	-0.62723	-3.06312	-2.55202	O	0.79420	2.95375	-4.32596
C	-0.52357	-2.41505	-3.91448	C	0.22452	5.61847	0.16603
C	-5.48915	-1.24203	0.83160	C	0.03190	5.14732	1.61332
C	-4.90065	-2.19563	1.87086	O	1.26586	4.72356	2.17100
C	-3.58163	-2.74574	1.36471	C	-1.01675	4.04144	1.67257
C	-3.70631	-3.30712	-0.04447	O	-1.36228	3.71471	2.99927
C	-4.46341	-2.38391	-1.00489	C	-2.27095	4.48710	0.94087
C	-4.89849	-3.09471	-2.27420	O	-3.15393	3.38305	0.94332
C	-4.27090	3.82365	0.06216	C	-1.92915	4.90653	-0.48593
C	-4.79145	3.45495	1.45950	O	-0.99626	5.98747	-0.41983
C	-4.49811	1.97555	1.67275	C	-3.14299	5.35514	-1.29005
C	-5.09646	1.16289	0.53226	O	-4.06585	6.11336	-0.53330
C	-4.70273	1.68825	-0.84648	C	-4.48553	3.57265	1.33140
C	-5.44310	0.97798	-1.98082	C	-4.82370	2.39889	2.24896
C	1.00402	4.84001	0.17058	O	-3.99045	2.44062	3.38122
C	0.32380	4.57964	1.51044	C	-4.70526	1.08856	1.46356
C	-0.94962	3.76177	1.30159	O	-5.08976	-0.01021	2.26024
C	-1.88824	4.43280	0.30321	C	-5.54104	1.20087	0.18911
C	-1.09272	4.75186	-0.96799	O	-5.27070	0.07834	-0.63407
C	-1.83806	5.60346	-1.97782	C	-5.19872	2.45181	-0.62230
C	4.52168	1.94646	-2.47458	O	-5.32930	3.61718	0.19888
C	4.92972	2.50310	-1.10550	C	-6.12956	2.64235	-1.80550
C	3.65094	2.65724	-0.28044	O	-7.48719	2.65054	-1.41458
C	2.69089	3.58347	-1.01872	C	-6.37193	-0.60578	-1.18907
C	2.45656	3.09281	-2.45431	C	-6.79356	-1.74988	-0.27172
C	1.61470	4.04718	-3.28124	O	-7.32536	-1.22338	0.93349
H	2.78366	-4.12945	-3.32605	C	-5.56705	-2.58681	0.02063
H	4.97110	-3.18295	-2.76887	O	-5.98092	-3.71210	0.77099
H	3.44198	-1.25202	-0.99523	C	-4.85622	-3.00077	-1.26521
H	5.47863	-0.43167	-0.63482	O	-3.51758	-3.34723	-0.92357
H	4.95852	-0.42953	-3.49476	C	-4.77160	-1.90998	-2.34300
H	1.94815	-0.66885	-3.02870	O	-5.97074	-1.14300	-2.41881
H	2.61410	0.95627	-4.78036	C	-4.56593	-2.49727	-3.73743
H	1.38131	-1.45913	-5.58981	O	-3.35823	-3.22801	-3.84131
H	3.49638	-0.33561	-5.62958	C	-3.20897	-4.70520	-0.79642
H	-2.81260	-5.22583	-1.40570	C	-2.55281	-4.92036	0.58280
H	-0.53941	-6.10026	-1.20745	O	-3.29161	-4.20205	1.55775
H	-0.41345	-6.01314	1.08698	C	-1.09192	-4.49848	0.60391
H	0.24269	-3.40747	-0.02154	O	-0.49081	-4.99088	1.77424
H	2.35759	-4.47607	-0.39035	C	-0.35604	-4.95077	-0.65203
H	0.85414	-4.60022	-2.77208	O	0.93187	-4.37950	-0.52411
H	-0.89876	-2.28263	-1.82915	C	-1.11013	-4.41848	-1.86938
H	-1.52596	-2.10077	-4.22975	O	-2.38442	-5.08925	-1.87167
H	-0.49847	-3.92918	-5.13796	C	-0.44246	-4.63129	-3.22591

H	0.09010	-1.51712	-3.82066	O	-0.55448	-3.50379	-4.07284
H	-6.47446	-0.88251	1.14484	C	2.06348	-5.11835	-0.88779
H	-5.61011	-3.02118	2.01734	C	3.04124	-5.05483	0.28339
H	-4.19515	-1.99425	3.66856	O	2.37690	-5.59143	1.41088
H	-2.86191	-1.91681	1.32527	C	3.45024	-3.60999	0.50301
H	-2.34397	-4.15217	1.85052	O	4.43698	-3.56869	1.51194
H	-4.21013	-4.28186	0.00026	C	3.96184	-3.00647	-0.80002
H	-3.81653	-1.54388	-1.28311	O	4.19887	-1.62721	-0.56539
H	-5.42558	-2.37240	-2.91370	C	2.94589	-3.17575	-1.92690
H	-6.45660	-3.90722	-1.47992	O	2.65557	-4.57471	-2.04046
H	-4.01028	-3.44076	-2.81062	C	3.46426	-2.76345	-3.29219
H	-4.43989	4.88744	-0.14394	O	4.64617	-3.45497	-3.63354
H	-5.87645	3.61761	1.45665	C	5.53488	-1.26196	-0.33723
H	-3.33457	3.98962	2.62516	C	5.54466	-0.19213	0.75054
H	-3.40530	1.85951	1.63626	O	4.94011	-0.72413	1.92051
H	-4.82515	0.58542	2.98671	C	4.79391	1.03513	0.26350
H	-6.18911	1.16147	0.62125	O	4.92593	2.06854	1.21023
H	-3.63117	1.53878	-1.00048	C	5.20300	1.46790	-1.14860
H	-5.49563	1.67491	-2.82352	O	4.11616	2.27250	-1.59206
H	-6.70485	-0.34693	-1.36021	C	5.34671	0.27219	-2.09851
H	-4.83450	0.12450	-2.29681	O	6.12494	-0.75892	-1.50859
H	1.84896	5.52564	0.28788	C	5.98377	0.58649	-3.44433
H	0.04470	5.54864	1.94089	O	5.24166	1.53841	-4.16976
H	1.77879	3.36417	1.91000	H	5.26172	3.44817	-2.84429
H	-0.67700	2.77931	0.89014	H	4.62180	5.57365	-1.71602
H	-1.06021	3.03096	3.09324	H	5.54096	5.06979	0.35818
H	-2.32440	5.34010	0.74454	H	3.09654	3.73117	0.16615
H	-0.84727	3.78672	-1.43249	H	2.74368	5.56614	1.30088
H	-2.10392	6.56137	-1.50595	H	1.94926	5.84508	-1.70014
H	-0.16511	6.05711	-2.84969	H	1.81542	2.80815	-1.96445
H	-2.75910	5.09333	-2.27478	H	1.30620	4.95585	-4.04836
H	5.89581	1.78349	0.42589	H	0.02847	4.29168	-3.00348
H	5.38702	1.76791	-3.12106	H	1.66232	2.56798	-4.51979
H	5.39615	3.48710	-1.25536	H	0.86548	6.50437	0.12975
H	3.19790	1.66682	-0.20550	H	-0.31284	5.99334	2.21637
H	4.12745	2.43501	1.60847	H	1.45383	3.81934	1.86483
H	3.11280	4.59564	-1.05818	H	-0.62082	3.15151	1.15119
H	1.92328	2.13542	-2.41489	H	-0.61315	3.18560	3.34119
H	0.57349	3.96218	-2.96778	H	-2.72139	5.34130	1.46242
H	2.80019	5.56415	-3.57701	H	-1.46473	4.05789	-1.00770
H	1.67370	3.73254	-4.33076	H	-3.62275	4.46622	-1.71305
O	4.09879	-3.73323	-0.96714	H	-2.77892	5.96406	-2.12468
O	5.50561	-1.29343	-1.09035	H	-4.80036	5.51835	-0.32532
O	3.80293	0.77185	-2.23611	H	-4.61341	4.52793	1.85130
O	2.84142	-2.24601	-4.01757	H	-5.85825	2.50737	2.59596
O	1.41658	-0.48794	-5.58067	H	-3.08860	2.67436	3.08875

O	-1.14573	-5.52058	0.69235	H	-3.65273	0.94535	1.17983
O	1.55672	-5.00493	-0.21139	H	-5.92862	-0.36391	1.91789
O	1.75449	-2.87523	-2.05921	H	-6.60530	1.23464	0.45967
O	-1.66433	-4.05091	-2.57346	H	-4.16483	2.35908	-0.98172
O	0.11197	-3.22544	-4.88484	H	-5.99470	1.80973	-2.50195
O	-4.72503	-1.45630	3.06391	H	-5.85566	3.57037	-2.32601
O	-3.12293	-3.73332	2.26013	H	-7.58952	3.35035	-0.75521
O	-2.35685	-3.45398	-0.45537	H	-7.20093	0.08440	-1.38633
O	-5.67772	-1.90563	-0.39648	H	-7.55184	-2.34775	-0.79476
O	-5.70091	-4.22228	-1.99416	H	-7.47835	-1.98625	1.51193
O	-4.24789	4.28416	2.45220	H	-4.87443	-1.95756	0.59772
O	-5.00934	1.53800	2.91088	H	-5.19857	-4.05774	1.23443
O	-4.59690	-0.17165	0.69112	H	-5.38507	-3.87587	-1.67408
O	-4.96931	3.09453	-0.91195	H	-3.92197	-1.26493	-2.10309
O	-6.75927	0.57494	-1.65080	H	-4.50965	-1.67938	-4.45926
O	1.18747	3.94209	2.42191	H	-5.43183	-3.12175	-3.99380
O	-1.61563	3.59811	2.53941	H	-3.37659	-3.99922	-3.25306
O	-2.89520	3.50337	-0.06762	H	-4.11307	-5.31752	-0.87392
O	0.10536	5.47408	-0.69927	H	-2.61396	-5.98083	0.84357
O	-1.05526	5.79384	-3.13675	H	-3.17206	-3.26217	1.32508
O	5.84686	1.60118	-0.52351	H	-1.05872	-3.39555	0.58094
O	3.89025	3.17209	1.01743	H	0.47471	-4.92525	1.66378
O	1.44974	3.59876	-0.32169	H	-0.29329	-6.04726	-0.69468
O	3.70465	2.89581	-3.12391	H	-1.26031	-3.33540	-1.74250
O	1.96195	5.40922	-3.12385	H	-0.87463	-5.53270	-3.68262
H	4.66169	-3.30116	-0.30598	H	0.62856	-4.79621	-3.08864
O	-2.38381	-0.08884	-2.40785	H	-1.49562	-3.27816	-4.16426
O	1.71132	1.18318	3.83031	H	1.80120	-6.15426	-1.12589
N	-1.84543	-0.12272	-0.20984	H	3.92679	-5.65222	0.02991
N	4.56379	0.51066	2.30697	H	3.02623	-5.67778	2.12064
N	4.23557	-0.17442	1.19781	H	2.55179	-3.05108	0.81099
N	3.67726	-1.28834	1.49244	H	4.58006	-2.63390	1.75001
N	3.63948	-1.36021	2.82742	H	4.89132	-3.50512	-1.10480
C	-1.56162	0.12631	-1.52224	H	2.02863	-2.61576	-1.69604
C	-0.18757	0.69478	-1.79090	H	3.69295	-1.69476	-3.29873
C	0.83875	0.12119	-0.81708	H	2.66339	-2.94086	-4.02460
C	0.37625	0.36288	0.58922	H	4.43751	-4.39796	-3.58657
C	1.21443	0.75094	1.61225	H	6.12937	-2.13015	-0.03652
C	0.74839	0.87953	2.92305	H	6.58696	0.07897	0.95837
C	-0.59137	0.62946	3.21163	H	4.26760	-0.09835	2.24475
C	-1.45816	0.27789	2.16946	H	3.72844	0.75057	0.20490
C	-0.98621	0.17273	0.86541	H	5.18087	2.88729	0.74731
C	1.34692	1.31585	5.19517	H	6.13762	2.04300	-1.10621
C	2.63466	1.33820	5.99013	H	4.33634	-0.10198	-2.29556
C	3.45269	0.04338	5.85720	H	6.07940	-0.36537	-3.98789
C	4.54208	0.02337	4.76742	H	6.99157	0.98892	-3.29445

C	4.19042	-0.24087	3.33736	H	4.29824	1.30717	-4.09412
C	2.99006	-2.51802	3.46810	O	2.51406	0.65605	-4.00369
C	1.47827	-2.28022	3.52623	O	-2.46773	-1.28731	1.41267
C	0.74612	-3.49013	4.10710	N	1.71064	0.21575	-1.93558
C	1.09696	-4.76294	3.33617	N	0.77853	1.94145	3.42082
C	2.60853	-4.99600	3.32339	N	1.85925	1.80456	2.64086
C	3.33187	-3.79892	2.70394	N	2.53569	0.76972	2.99223
H	-2.81823	-0.32302	0.01075	N	1.88880	0.21118	4.02368
H	-0.25215	1.78226	-1.64389	C	1.57362	0.30929	-3.28256
H	0.05597	0.51710	-2.84194	C	0.20919	-0.06088	-3.81374
H	1.82783	0.54732	-0.98983	C	-0.89250	0.45202	-2.88102
H	0.93979	-0.96176	-0.97933	C	-0.65590	-0.06075	-1.48338
H	2.26027	0.94886	1.42413	C	-1.67469	-0.41841	-0.61084
H	-0.97927	0.69526	4.22139	C	-1.38554	-0.89829	0.66980
H	-2.50405	0.08037	2.38934	C	-0.05828	-0.98485	1.09535
H	0.73484	0.45767	5.50578	C	0.97250	-0.62774	0.22567
H	0.75796	2.23134	5.33559	C	0.67535	-0.16899	-1.05198
H	2.36732	1.48863	7.04012	C	-2.27282	-1.51829	2.81031
H	3.23547	2.20258	5.68645	C	-1.89666	-0.24470	3.56101
H	2.77420	-0.81113	5.74820	C	-1.12350	-0.54559	4.84891
H	3.98498	-0.12049	6.79760	C	-0.25547	0.64058	5.30351
H	5.27661	-0.74536	5.03520	C	0.79574	0.94466	4.29051
H	5.08061	0.97470	4.77148	C	2.42207	-0.98258	4.70232
H	3.40554	-2.57513	4.48072	C	2.36921	-2.18983	3.76526
H	1.12693	-2.08245	2.50334	C	2.98655	-3.42209	4.42716
H	1.27361	-1.38003	4.11330	C	4.40266	-3.13919	4.92688
H	-0.33220	-3.30429	4.07509	C	4.40801	-1.95262	5.89202
H	1.02220	-3.61127	5.16278	C	3.83669	-0.70391	5.21710
H	0.75407	-4.65676	2.29865	H	2.59434	0.53378	-1.55193
H	0.57910	-5.62493	3.76896	H	0.15969	-1.15722	-3.88322
H	2.85178	-5.89552	2.75056	H	0.12368	0.35985	-4.81541
H	2.96686	-5.15908	4.34816	H	-1.87512	0.13821	-3.24248
H	4.41672	-3.94492	2.69615	H	-0.86994	1.54967	-2.88410
H	3.00086	-3.68627	1.66575	H	-2.71962	-0.32042	-0.89153
				H	0.18919	-1.34282	2.08767
				H	2.01051	-0.72445	0.53902
				H	-1.51934	-2.30995	2.94020
				H	-3.23204	-1.90702	3.15677
				H	-2.80049	0.33608	3.76780
				H	-1.28109	0.37805	2.90062
				H	-0.47250	-1.41775	4.69783
				H	-1.80592	-0.81172	5.66006
				H	0.21057	0.43346	6.27047
				H	-0.86692	1.53951	5.41743
				H	1.75580	-1.15017	5.55560
				H	2.92408	-1.94232	2.85150

H	1.33334	-2.40206	3.47721
H	2.98666	-4.23564	3.69921
H	2.35473	-3.73476	5.26807
H	5.04748	-2.91093	4.06808
H	4.81477	-4.02827	5.41285
H	5.42255	-1.74107	6.24050
H	3.80875	-2.19716	6.77851
H	3.81041	0.14607	5.90521
H	4.47752	-0.42984	4.37133

HPβ-CD_mol1:CIL_K02

Atom	x [Å]	y [Å]	z [Å]
C	-6.39229	0.58487	-0.41725
C	-6.56915	1.46067	-1.64977
O	-6.82431	0.62749	-2.76569
C	-5.30065	2.25600	-1.87769
O	-5.56114	3.14081	-2.94449
C	-4.83523	2.98763	-0.62647
O	-3.49338	3.40137	-0.88252
C	-4.85710	2.08455	0.61304
O	-6.10919	1.40015	0.69147
C	-4.75703	2.85060	1.92848
O	-3.53657	3.53878	2.07231
C	-3.23719	4.75993	-1.07109
C	-2.43276	4.95288	-2.37087
O	-3.07564	4.31048	-3.45159
C	-0.99199	4.48236	-2.21992
O	-0.27636	4.85341	-3.37232
C	-0.39374	5.06656	-0.95046
O	0.92275	4.55905	-0.83994
C	-1.26250	4.65391	0.23378
O	-2.54172	5.28008	0.05178
C	-0.66284	5.03081	1.56784
O	-0.16419	6.34853	1.53927
C	0.52959	6.65636	2.73171
C	1.35980	7.91371	2.54184
C	2.00839	8.32807	3.85732
O	2.33783	7.73393	1.52823
C	1.94458	5.45283	-0.46164
C	2.95976	5.53036	-1.59920
O	2.28453	5.95231	-2.76911
C	3.60483	4.17630	-1.80980
O	4.58383	4.31601	-2.80891
C	4.18043	3.67738	-0.49760
O	4.64631	2.35669	-0.73261
C	3.10555	3.66905	0.58535
O	2.58091	5.00209	0.71431

HPβ-CD:CIL_mol2_K02

Atom	x [Å]	y [Å]	z [Å]
C	6.12396	0.77507	0.45825
C	6.66766	0.38187	-0.91746
O	6.61114	1.49169	-1.79929
C	5.88393	-0.78596	-1.48097
O	6.54425	-1.22222	-2.64441
C	5.66028	-1.91100	-0.48092
O	4.57739	-2.68384	-0.98618
C	5.20979	-1.37773	0.87427
O	6.08822	-0.34166	1.31311
C	5.21235	-2.45249	1.94397
O	4.40605	-2.05905	3.03614
C	3.12393	-2.64926	3.07219
C	3.11681	-3.87707	3.96771
C	1.73852	-4.51710	4.02998
O	4.08404	-4.77354	3.43882
C	4.83834	-3.91705	-1.59058
C	4.13442	-3.93226	-2.95217
O	4.56491	-2.84717	-3.75181
C	2.62795	-3.89237	-2.75123
O	1.99043	-3.95925	-4.00179
C	2.15288	-4.96742	-1.77346
O	0.79695	-4.64081	-1.48411
C	2.97988	-4.89118	-0.48452
O	4.37085	-4.96813	-0.78337
C	2.74381	-6.01565	0.51760
O	1.37956	-6.26180	0.80196
C	-0.19288	-5.64693	-1.50337
C	-0.97017	-5.58598	-2.81583
O	-0.06716	-5.82716	-3.88149
C	-1.63714	-4.23194	-2.96136
O	-2.41297	-4.26009	-4.13506
C	-2.48888	-3.98728	-1.73083
O	-3.04729	-2.68887	-1.82448
C	-1.68654	-4.13529	-0.43834
O	-1.05728	-5.42392	-0.42117

C	3.63166	3.17221	1.92963	C	-2.59202	-4.02218	0.77223
O	4.97867	3.52208	2.18158	O	-3.72964	-4.82031	0.55950
C	5.98822	2.04661	-0.48923	C	-4.77724	-4.56997	1.46827
C	6.51460	1.27588	-1.69591	C	-6.00127	-5.34034	1.01648
O	6.41244	2.10086	-2.83973	C	-7.13734	-5.18534	2.01834
C	5.69871	0.01247	-1.86566	O	-6.36233	-4.82246	-0.25675
O	6.25027	-0.71312	-2.94071	C	-4.44597	-2.60145	-1.69477
C	5.71226	-0.79316	-0.57717	C	-4.97496	-1.75663	-2.85338
O	4.73938	-1.81883	-0.72254	O	-4.50891	-2.30890	-4.06940
C	5.38097	0.03361	0.67012	C	-4.53940	-0.31208	-2.70164
O	6.11861	1.26902	0.67692	O	-5.12408	0.41661	-3.75411
C	5.76404	-0.70989	1.93898	C	-4.98921	0.18787	-1.34080
O	7.15279	-0.98795	1.91670	O	-4.54039	1.52491	-1.21653
C	7.52059	-2.33994	2.12231	C	-4.40402	-0.69471	-0.24015
C	7.96250	-2.58580	3.56048	O	-4.81676	-2.04099	-0.46400
C	6.83333	-2.36384	4.55526	C	-4.90725	-0.31305	1.13699
O	8.41530	-3.93287	3.57660	O	-6.31715	-0.32217	1.10189
C	5.18585	-3.14306	-0.76361	C	-6.97481	-0.66493	2.30315
C	4.49972	-3.86569	-1.91836	C	-7.29780	0.54712	3.16640
O	4.73223	-3.13851	-3.10916	C	-8.15097	0.15206	4.36391
C	3.00800	-4.03186	-1.67084	O	-6.11201	1.20229	3.59640
O	2.51713	-4.84551	-2.71276	C	-5.46565	2.51039	-0.85107
C	2.75591	-4.63174	-0.29467	C	-5.29412	3.68364	-1.81828
O	1.35190	-4.56388	-0.06037	O	-5.49446	3.21502	-3.13755
C	3.51966	-3.84625	0.77300	C	-3.89873	4.25178	-1.65387
O	4.90722	-3.81741	0.43514	O	-3.76122	5.34962	-2.52528
C	3.43913	-4.48727	2.14590	C	-3.64902	4.63290	-0.20360
O	4.01464	-3.64022	3.11863	O	-2.27385	4.97048	-0.13192
C	3.08121	-2.99658	3.96255	C	-3.93707	3.44177	0.70881
C	2.12650	-2.04691	3.25423	O	-5.25523	2.94929	0.46902
C	1.18166	-1.42134	4.27077	C	-3.86175	3.78934	2.18237
O	2.92356	-1.06839	2.60678	O	-3.96647	2.63868	2.99080
C	0.73711	-5.68557	0.52829	C	-1.88135	6.20130	0.40195
C	-0.15612	-6.37627	-0.49632	C	-0.78985	6.74721	-0.51489
O	0.61981	-6.68933	-1.63818	O	-1.34272	6.88725	-1.81046
C	-1.30586	-5.46797	-0.88022	C	0.39046	5.78949	-0.50630
O	-2.11630	-6.19544	-1.77197	O	1.40200	6.32297	-1.32961
C	-2.06695	-4.97921	0.34094	C	0.85205	5.57289	0.92176
O	-2.95591	-3.95558	-0.10761	O	1.84878	4.56750	0.86858
C	-1.09371	-4.38710	1.36690	C	-0.30776	5.11187	1.80438
O	-0.03167	-5.30478	1.63721	O	-1.38314	6.05120	1.71087
C	-1.72825	-4.13551	2.73878	C	0.06796	5.03346	3.27220
O	-2.91204	-3.36265	2.69646	O	0.56818	6.26761	3.74680
C	-4.31922	-4.26594	-0.20329	C	2.94048	4.71162	1.74329
C	-4.85810	-3.82870	-1.56687	C	4.19632	4.93033	0.90121
O	-4.08804	-4.43944	-2.58342	O	3.96051	6.02618	0.03592

C	-4.82826	-2.32114	-1.70501	C	4.52953	3.67687	0.11553
O	-5.43855	-1.99969	-2.93106	O	5.76778	3.90384	-0.51687
C	-5.55135	-1.68821	-0.53200	C	4.58517	2.49167	1.06347
O	-5.33366	-0.29746	-0.66477	O	4.84957	1.32897	0.29669
C	-4.96717	-2.19147	0.78455	C	3.27542	2.35453	1.83343
O	-5.04232	-3.62431	0.82975	O	3.06830	3.57516	2.55319
C	-5.67480	-1.61909	1.99584	C	3.30562	1.22806	2.84596
O	-7.05542	-1.90076	1.92206	O	4.49782	1.32648	3.59171
C	-7.88647	-0.92464	2.53412	C	4.49392	0.55859	4.78268
C	-7.68938	-0.75091	4.04040	C	5.94544	0.33657	5.17038
C	-7.60858	-2.08087	4.77632	C	6.05591	-0.48277	6.44412
O	-6.57871	0.08793	4.32089	O	6.63073	-0.34946	4.14090
H	-7.30828	0.03512	-0.17335	H	6.79764	1.49410	0.93538
H	-7.41035	2.14490	-1.47132	H	7.71701	0.08834	-0.79238
H	-6.69394	1.18578	-3.54688	H	5.89460	1.32676	-2.44042
H	-4.51004	1.54455	-2.14688	H	4.87513	-0.41582	-1.70836
H	-4.71950	3.56824	-3.19731	H	5.96085	-1.84726	-3.11280
H	-5.48014	3.86228	-0.45314	H	6.56655	-2.52441	-0.37717
H	-4.03161	1.36295	0.54173	H	4.18848	-0.98587	0.75914
H	-4.82506	2.12641	2.74811	H	6.23658	-2.58505	2.30966
H	-5.62066	3.52592	2.00751	H	4.86783	-3.40096	1.51598
H	-3.48361	4.26608	1.43281	H	5.91536	-4.07616	-1.70895
H	-4.17156	5.32803	-1.12811	H	4.40801	-4.85473	-3.47552
H	-2.40882	6.02914	-2.58190	H	4.01826	-2.05508	-3.57245
H	-2.63608	3.44822	-3.63258	H	2.39933	-2.92542	-2.26823
H	-0.99760	3.38271	-2.10545	H	1.21134	-4.54402	-3.93541
H	0.65226	5.01916	-3.12038	H	2.22844	-5.96327	-2.23254
H	-0.37745	6.16202	-1.01421	H	2.74531	-3.91957	-0.01943
H	-1.39701	3.56250	0.24150	H	3.30100	-5.76854	1.43080
H	0.16617	4.33261	1.75788	H	3.15527	-6.93966	0.09750
H	-1.41244	4.89819	2.36224	H	0.87249	-5.43920	0.75718
H	1.53345	6.43413	-0.22130	H	0.25624	-6.63095	-1.34894
H	3.73381	6.25793	-1.31717	H	-1.74332	-6.36552	-2.79114
H	2.90623	5.83771	-3.50306	H	-0.57032	-5.71943	-4.70259
H	2.81572	3.47042	-2.12151	H	-0.85081	-3.46402	-3.02681
H	5.06032	3.46901	-2.89437	H	-2.98479	-3.46944	-4.14185
H	5.00642	4.32429	-0.17784	H	-3.28617	-4.73921	-1.71804
H	2.28420	3.01041	0.28406	H	-0.92062	-3.35226	-0.37124
H	3.49420	2.08411	1.95995	H	-2.87736	-2.96457	0.88852
H	3.00470	3.60811	2.71702	H	-2.04182	-4.33800	1.67326
H	5.52050	2.75914	1.92801	H	-4.89670	-3.59791	-1.69113
H	6.57420	2.95610	-0.31852	H	-6.07340	-1.78785	-2.81715
H	7.56297	1.00996	-1.50214	H	-4.69824	-1.65977	-4.76181
H	6.49945	1.51706	-3.60778	H	-3.43841	-0.26270	-2.74550
H	4.66148	0.29999	-2.08837	H	-5.02970	1.36781	-3.56021
H	5.66765	-1.47292	-3.12145	H	-6.08385	0.14355	-1.27985

H	6.71394	-1.22481	-0.44794	H	-3.30427	-0.61969	-0.25100
H	4.30227	0.24368	0.68970	H	-4.51143	0.66914	1.42736
H	5.16456	-1.62401	2.01366	H	-4.53435	-1.06137	1.85251
H	5.51709	-0.07866	2.80238	H	-6.48624	2.11640	-0.88108
H	6.27408	-3.17944	-0.88636	H	-6.03635	4.45279	-1.56376
H	4.94333	-4.86843	-1.98553	H	-5.15694	3.90188	-3.73075
H	4.30628	-3.61713	-3.83335	H	-3.18086	3.45770	-1.91014
H	2.53407	-3.03535	-1.69719	H	-2.89628	5.76800	-2.35850
H	1.75648	-5.35846	-2.38073	H	-4.27659	5.49238	0.07326
H	3.09175	-5.67929	-0.30077	H	-3.20612	2.65131	0.48298
H	3.13084	-2.81981	0.82249	H	-4.65482	4.51260	2.41803
H	4.00984	-5.42373	2.12731	H	-2.89929	4.26576	2.38885
H	2.39415	-4.71913	2.39152	H	-4.87735	2.28202	2.94410
H	1.49302	-6.37966	0.91081	H	-2.72789	6.89212	0.46958
H	-0.55484	-7.29246	-0.03950	H	-0.46422	7.72215	-0.12756
H	-0.00535	-6.98978	-2.31559	H	-0.61800	7.10669	-2.41316
H	-0.88685	-4.57411	-1.36261	H	0.05748	4.81492	-0.88853
H	-2.75343	-5.58127	-2.18326	H	2.26387	6.01192	-0.99463
H	-2.62910	-5.81033	0.79178	H	1.26608	6.50903	1.31924
H	-0.67405	-3.46311	0.95159	H	-0.63873	4.12270	1.45810
H	-1.01371	-3.58707	3.35953	H	0.85773	4.28763	3.40111
H	-1.90923	-5.11089	3.20784	H	-0.81173	4.70803	3.84410
H	-3.62156	-3.84229	2.24157	H	-0.11053	6.93250	3.56671
H	-4.48213	-5.33891	-0.06014	H	2.77182	5.55095	2.42682
H	-5.90344	-4.16246	-1.62620	H	5.03415	5.14747	1.57845
H	-4.30722	-4.00041	-3.41782	H	4.71617	6.06335	-0.56866
H	-3.78057	-1.97737	-1.68440	H	3.73127	3.49573	-0.62302
H	-5.60098	-1.03854	-2.94073	H	6.00871	3.10608	-1.02687
H	-6.62170	-1.92516	-0.57837	H	5.38238	2.65059	1.79931
H	-3.91570	-1.87897	0.82853	H	2.44014	2.18426	1.14000
H	-5.49415	-0.53609	2.01708	H	3.25270	0.25876	2.32896
H	-5.23973	-2.05454	2.90914	H	2.42383	1.32389	3.49644
H	2.49238	-3.74215	4.51687	H	2.42321	-1.90535	3.47242
H	3.67686	-2.41957	4.67860	H	2.78524	-2.91250	2.05772
H	1.53493	-2.60765	2.50915	H	3.42469	-3.55252	4.97328
H	0.48897	-0.74229	3.77213	H	1.74031	-5.37152	4.71262
H	0.59837	-2.18668	4.79167	H	0.99256	-3.80225	4.39029
H	1.75317	-0.85271	5.01099	H	1.44483	-4.87962	3.03853
H	2.37223	-0.53428	2.01961	H	4.11640	-5.55270	4.00750
H	6.69062	-3.01685	1.87729	H	3.95073	1.09026	5.57760
H	8.36010	-2.56197	1.45302	H	4.01969	-0.41430	4.60626
H	8.79273	-1.89862	3.78475	H	6.40241	1.32803	5.32974
H	7.16419	-2.59661	5.57249	H	7.10634	-0.66545	6.68165
H	6.50723	-1.32006	4.54126	H	5.59388	0.03554	7.28833
H	5.98427	-3.00463	4.29728	H	5.56118	-1.44916	6.30612
H	8.70260	-4.13904	4.47387	H	6.22536	-0.10692	3.29113

H	-0.17805	6.78873	3.56332	H	-7.90829	-1.16503	2.02203
H	1.20451	5.82344	2.99357	H	-6.36754	-1.37505	2.88829
H	0.70323	8.71475	2.18288	H	-7.82853	1.27455	2.54207
H	2.63574	9.20705	3.69768	H	-8.36120	1.02954	4.97849
H	1.25693	8.56707	4.61445	H	-9.09895	-0.29041	4.04780
H	2.64055	7.51957	4.23977	H	-7.61536	-0.58011	4.97841
H	2.73979	6.85908	1.64731	H	-5.64043	0.60927	4.21940
H	-7.72794	0.05546	2.06231	H	-4.49543	-4.88548	2.48523
H	-8.91069	-1.25544	2.33741	H	-5.01884	-3.49548	1.47517
H	-8.57537	-0.20408	4.39185	H	-5.72224	-6.40053	0.92644
H	-7.50371	-1.91090	5.85033	H	-8.02890	-5.71272	1.66876
H	-8.50965	-2.67442	4.60200	H	-6.86166	-5.59503	2.99360
H	-6.75070	-2.66581	4.43055	H	-7.38865	-4.12710	2.13812
H	-5.79612	-0.43806	4.55246	H	-7.09037	-5.35625	-0.59723
O	-4.01330	-0.58245	5.20087	O	-4.66580	-0.66528	4.97476
O	1.77109	0.81382	0.55091	O	1.39229	-1.13153	0.39072
N	-2.86962	-0.51415	3.25778	N	-2.87388	0.02217	3.77074
N	-2.13595	1.68003	-3.95903	N	3.39977	-0.14427	-3.56847
N	-3.13833	0.78737	-3.88564	N	4.08305	1.00160	-3.39010
N	-2.67765	-0.40812	-3.89448	N	3.32786	2.02099	-3.58269
N	-1.34405	-0.31558	-3.98678	N	2.12013	1.54836	-3.90007
C	-3.11184	-0.08558	4.52972	C	-3.62310	-0.93356	4.37034
C	-2.16880	0.96709	5.06961	C	-3.14469	-2.35829	4.20352
C	-1.57501	1.86659	3.98230	C	-1.63210	-2.46890	4.02031
C	-1.05143	1.03922	2.83836	C	-1.15873	-1.50001	2.96841
C	0.10453	1.34311	2.12773	C	-0.11724	-1.76523	2.08634
C	0.54696	0.51315	1.09619	C	0.32877	-0.79329	1.18590
C	-0.18371	-0.61435	0.72957	C	-0.29466	0.45610	1.15080
C	-1.34442	-0.91945	1.42963	C	-1.36057	0.71758	2.00754
C	-1.75271	-0.12403	2.49682	C	-1.79257	-0.25322	2.90665
C	1.99010	0.49729	-0.82707	C	1.38806	-0.63028	-0.94962
C	0.97336	1.15968	-1.74268	C	0.22166	-1.23744	-1.72860
C	1.41144	1.06112	-3.20243	C	-0.10038	-0.62728	-3.09292
C	0.35173	1.60194	-4.16988	C	1.01807	-0.71727	-4.14681
C	-1.00827	0.99058	-4.03298	C	2.16366	0.20174	-3.88500
C	-0.49657	-1.52578	-4.00908	C	1.00486	2.45353	-4.21478
C	-1.33801	-2.78692	-3.81605	C	0.44799	3.11013	-2.95097
C	-0.41728	-4.00887	-3.82101	C	-0.76251	3.96575	-3.33000
C	0.41712	-4.10415	-5.09639	C	-0.35825	5.04273	-4.33615
C	1.22569	-2.82312	-5.29221	C	0.25034	4.41096	-5.59093
C	0.31129	-1.59535	-5.30761	C	1.43479	3.50326	-5.24138
H	-3.24574	-1.43551	3.04030	H	-3.23179	0.97852	3.76191
H	-1.37129	0.41222	5.58182	H	-3.66008	-2.74770	3.31398
H	-2.70469	1.54072	5.82666	H	-3.50325	-2.93237	5.05863
H	-0.77726	2.48619	4.40002	H	-1.35555	-3.49429	3.76180
H	-2.34471	2.54383	3.59492	H	-1.13443	-2.23235	4.96976

H	0.70816	2.20466	2.39986	H	0.37038	-2.73674	2.08827
H	0.14999	-1.26444	-0.07388	H	0.02539	1.22538	0.45670
H	-1.91783	-1.80246	1.17219	H	-1.86556	1.67828	1.98835
H	2.03348	-0.59127	-0.96827	H	2.35806	-0.93888	-1.35030
H	2.98334	0.90277	-1.02741	H	1.36692	0.46863	-0.95843
H	0.87998	2.21498	-1.45221	H	-0.68431	-1.14657	-1.11575
H	-0.01612	0.70388	-1.60949	H	0.41926	-2.30975	-1.83446
H	1.67718	0.02628	-3.44944	H	-0.41935	0.41384	-2.96636
H	2.32718	1.64336	-3.35551	H	-0.97104	-1.15819	-3.49334
H	0.68693	1.48077	-5.20501	H	0.60665	-0.48146	-5.13382
H	0.21389	2.67701	-4.01034	H	1.41121	-1.73939	-4.19837
H	0.18524	-1.43804	-3.15329	H	0.23597	1.81200	-4.65982
H	-2.07011	-2.85910	-4.62882	H	1.22989	3.74248	-2.50754
H	-1.89843	-2.73420	-2.87652	H	0.17650	2.35093	-2.20939
H	-1.01533	-4.91410	-3.67367	H	-1.19148	4.41972	-2.43428
H	0.27269	-3.93391	-2.97101	H	-1.54015	3.32260	-3.76653
H	-0.24295	-4.25748	-5.96010	H	0.38728	5.69798	-3.86340
H	1.08799	-4.96579	-5.03560	H	-1.22403	5.65936	-4.59949
H	1.80088	-2.85967	-6.22162	H	0.58150	5.18265	-6.29132
H	1.94749	-2.73053	-4.46788	H	-0.51794	3.81996	-6.10580
H	0.90362	-0.68699	-5.44697	H	1.82465	3.00601	-6.13447
H	-0.39343	-1.65766	-6.14619	H	2.24681	4.09960	-4.81022

	SBEβ-CD_mol1:CIL_K03				*SBEβ-CD:CIL_mol2_K03*		
**Atom**	**x [Å]**	**y [Å]**	**z [Å]**	**Atom**	**x [Å]**	**y [Å]**	**z [Å]**
C	4.22696	-1.95167	-1.28024	C	2.84543	-5.39865	-1.43745
C	3.98322	-2.87676	-2.47550	C	2.07943	-6.57226	-2.03720
O	4.36972	-2.22800	-3.67613	O	2.68373	-6.91550	-3.26912
C	2.51368	-3.24594	-2.50313	C	0.62638	-6.18234	-2.24947
O	2.28699	-4.08584	-3.61211	O	-0.04810	-7.34911	-2.67128
C	2.14349	-3.92528	-1.19655	C	0.01823	-5.58326	-0.98506
O	0.72898	-4.06945	-1.20049	O	-1.24256	-5.01342	-1.32032
C	2.57630	-3.10237	0.02005	C	0.91862	-4.48830	-0.41281
O	3.93014	-2.65005	-0.10729	O	2.23574	-5.00458	-0.23384
C	2.51716	-3.93698	1.28890	C	0.43771	-3.99862	0.93558
O	2.78985	-3.19181	2.44836	O	1.17853	-2.85218	1.30853
C	0.17632	-5.33589	-0.97972	C	1.58129	-2.83181	2.67164
C	-0.89509	-5.57620	-2.04705	C	2.85464	-3.63422	2.90411
O	-0.31314	-5.36176	-3.31828	C	3.33943	-3.51385	4.34803
C	-2.09860	-4.67513	-1.82700	C	4.62681	-4.29220	4.58029
O	-3.07702	-5.05222	-2.77032	S	5.26698	-4.10262	6.24855
C	-2.56623	-4.82370	-0.39069	O	5.64434	-2.66762	6.36575
O	-3.64794	-3.94110	-0.15281	O	4.15935	-4.47968	7.15501
C	-1.42213	-4.49365	0.55789	O	6.43386	-5.00895	6.32765
O	-0.37868	-5.44119	0.31001	C	-2.36975	-5.82355	-1.08346
C	-1.85048	-4.57256	2.01838	C	-3.24948	-5.79590	-2.34111
O	-2.67152	-5.68598	2.30919	O	-2.48715	-6.16368	-3.48168

C	-4.91894	-4.51174	-0.07394	C	-3.90703	-4.43839	-2.52063
C	-5.88792	-3.64143	-0.89049	O	-4.82434	-4.50906	-3.57921
O	-5.27008	-3.27852	-2.11631	C	-4.57560	-4.00943	-1.22315
C	-6.32860	-2.40305	-0.13042	O	-5.01606	-2.68042	-1.43616
O	-7.49459	-1.89462	-0.74333	C	-3.59054	-4.06540	-0.06046
C	-6.56637	-2.61711	1.36475	O	-3.07145	-5.39623	0.05163
O	-6.74044	-1.27186	1.78789	C	-4.24033	-3.65291	1.25262
C	-5.38887	-3.37460	1.98388	O	-5.55732	-4.13549	1.41771
O	-5.30545	-4.63044	1.27589	C	-5.64897	-5.52783	1.67333
C	-5.54931	-3.79472	3.43785	C	-7.10918	-5.86882	1.90192
O	-6.77723	-4.45918	3.65491	C	-7.30684	-7.36054	2.15917
C	-6.90031	-0.93098	3.12940	C	-8.77370	-7.72180	2.34147
C	-7.70152	0.37334	3.15183	S	-9.01513	-9.48049	2.61319
O	-8.94358	0.16639	2.52366	O	-8.45606	-10.14919	1.41339
C	-6.89599	1.45372	2.44673	O	-8.27211	-9.79991	3.85620
O	-7.59827	2.67204	2.56680	O	-10.48079	-9.65583	2.74863
C	-5.51912	1.54982	3.07728	C	-6.30650	-2.30848	-1.01952
O	-4.78534	2.52680	2.35203	C	-7.03245	-1.74317	-2.24002
C	-4.80856	0.20246	3.05879	O	-7.07267	-2.74240	-3.24221
O	-5.64954	-0.72651	3.75907	C	-6.30320	-0.50937	-2.73646
C	-3.43294	0.23976	3.71187	O	-7.04115	0.02491	-3.81083
O	-3.36692	1.00841	4.90664	C	-6.15852	0.48361	-1.59861
C	-4.43152	3.67597	3.05429	O	-5.33166	1.53230	-2.07367
C	-4.55215	4.87750	2.12680	C	-5.51552	-0.16508	-0.37617
O	-5.86573	4.89840	1.61090	O	-6.24868	-1.33998	-0.00495
C	-3.51469	4.80235	1.02338	C	-5.45310	0.79724	0.80387
O	-3.62740	5.98995	0.27384	O	-6.60587	1.60134	0.94571
C	-2.12902	4.63581	1.63846	C	-7.74850	0.90691	1.41712
O	-1.19588	4.45054	0.58808	C	-8.86391	1.91492	1.61844
C	-2.09846	3.41375	2.55534	C	-10.14847	1.24722	2.10264
O	-3.11573	3.56514	3.56210	C	-11.28343	2.24574	2.27601
C	-0.78678	3.26048	3.30351	S	-12.80388	1.46372	2.82483
O	-0.68138	2.01854	3.96531	O	-13.15025	0.47896	1.77136
C	-0.47527	5.58795	0.17804	O	-12.48084	0.83219	4.12704
C	-0.49843	5.64882	-1.35681	O	-13.78873	2.56576	2.93964
O	-1.82821	5.55350	-1.83043	C	-5.76993	2.85423	-1.92077
C	0.38626	4.57754	-1.98130	C	-5.64792	3.53888	-3.27506
O	0.59753	4.91620	-3.33394	O	-6.40677	2.81788	-4.22772
C	1.71304	4.42366	-1.24992	C	-4.18991	3.60789	-3.69571
O	2.28117	3.17927	-1.64077	O	-4.17519	4.28825	-4.92558
C	1.52249	4.37103	0.25444	C	-3.36054	4.29152	-2.61493
O	0.83871	5.54916	0.67707	O	-1.98594	4.17599	-2.97810
C	2.84657	4.21691	0.99286	C	-3.60522	3.60393	-1.26586
O	3.94135	4.87504	0.38466	O	-5.00062	3.54860	-0.97825
C	3.45932	3.12760	-2.39234	C	-2.96909	4.35773	-0.12350
C	3.18553	2.17622	-3.56181	O	-3.04447	3.54328	1.02637

O	2.10807	2.76906	-4.25645	C	-2.39962	4.10279	2.15618
C	1.76938	2.29887	-5.56267	C	-3.13507	5.28474	2.77766
C	0.43909	2.95379	-5.90558	C	-2.24129	5.99814	3.79325
C	-0.67641	2.49978	-4.95548	C	-2.78857	7.36849	4.16915
C	-1.83634	3.48366	-4.90604	S	-1.57914	8.35679	5.04884
S	-3.11051	2.97978	-3.75054	O	-1.20645	7.57576	6.25339
O	-4.15906	4.01749	-3.81896	O	-0.44611	8.53154	4.10166
O	-3.56051	1.63418	-4.17112	O	-2.26733	9.63068	5.36837
O	-2.42999	2.94953	-2.41090	C	-1.23641	5.36591	-3.08008
C	2.82168	0.80709	-2.98979	C	-0.72157	5.53140	-4.50742
O	2.58806	-0.13989	-4.00401	O	-1.80372	5.56452	-5.41629
C	3.90804	0.33668	-2.02406	C	0.22143	4.39825	-4.84289
O	3.40625	-0.80534	-1.33882	O	0.63901	4.58512	-6.17356
C	4.28159	1.39699	-0.97942	C	1.38810	4.43407	-3.87601
O	4.53172	2.66517	-1.60033	O	2.18179	3.29075	-4.14659
C	5.50807	0.99540	-0.18504	C	0.92112	4.42762	-2.41086
O	6.53884	0.62433	-1.07425	O	-0.14133	5.35926	-2.19659
C	7.75813	0.20657	-0.46911	C	2.09019	4.87003	-1.53136
C	7.88858	-1.31609	-0.43490	O	2.00508	4.55225	-0.16281
C	7.16380	-1.96244	0.74771	C	1.02042	5.24416	0.59870
C	6.96793	-3.46312	0.58913	C	1.27145	6.75879	0.73675
S	6.45945	-4.22539	2.13548	C	0.19991	7.62940	0.06814
O	5.87530	-5.53573	1.77562	C	-1.12421	7.53267	0.81818
O	7.68303	-4.31672	2.96149	S	-2.50119	8.27944	-0.04962
O	5.45928	-3.29570	2.74569	O	-2.70102	7.47337	-1.28527
H	5.28153	-1.65507	-1.22039	O	-3.65153	8.17215	0.88471
H	4.59415	-3.77877	-2.32917	O	-2.10294	9.67806	-0.33201
H	4.11528	-2.82157	-4.39939	C	3.54130	3.45644	-4.44714
H	1.93646	-2.31471	-2.59148	C	3.84935	2.68143	-5.73053
H	1.35349	-4.36736	-3.58690	O	3.00797	3.15454	-6.76423
H	2.62505	-4.91439	-1.16954	C	3.65482	1.19597	-5.49578
H	1.90279	-2.23801	0.10364	O	4.04791	0.51715	-6.66585
H	3.23417	-4.76487	1.17772	C	4.48404	0.77213	-4.29815
H	1.51042	-4.36333	1.36514	O	4.21340	-0.60380	-4.09974
H	3.76477	-3.21830	2.56341	C	4.11864	1.60987	-3.07520
H	0.94964	-6.10888	-1.03875	O	4.34961	2.98696	-3.39676
H	-1.23104	-6.61848	-1.95161	C	4.91784	1.25586	-1.84190
H	-1.01789	-5.46565	-3.97286	O	6.29163	1.18825	-2.15185
H	-1.79761	-3.62645	-1.97289	C	7.05774	0.58037	-1.11845
H	-3.86024	-4.48144	-2.65580	C	7.12765	1.41131	0.15665
H	-2.87080	-5.86203	-0.20153	C	7.98303	0.72794	1.22331
H	-1.04854	-3.47786	0.35918	C	7.95610	1.50455	2.53110
H	-2.34983	-3.62955	2.27529	S	9.05895	0.84251	3.78038
H	-0.95185	-4.65363	2.63708	O	8.76176	1.62761	5.00745
H	-3.57248	-5.47969	2.01348	O	8.73873	-0.59897	3.91444
H	-4.90995	-5.53225	-0.47115	O	10.43197	1.07997	3.26677

H	-6.77108	-4.24002	-1.13275	C	5.30375	-1.45474	-3.88932
H	-4.59738	-2.60555	-1.88959	C	5.10081	-2.70088	-4.75221
H	-5.51097	-1.67048	-0.18097	O	4.92987	-2.27674	-6.09064
H	-7.75977	-1.12866	-0.21382	C	3.90169	-3.49070	-4.25909
H	-7.48681	-3.19029	1.53997	O	3.83084	-4.69244	-4.99158
H	-4.46375	-2.79775	1.84524	C	4.03563	-3.75889	-2.77077
H	-5.51792	-2.92529	4.09259	O	2.81607	-4.36290	-2.38341
H	-4.69318	-4.43895	3.68761	C	4.26593	-2.46191	-2.00673
H	-6.79871	-5.21470	3.05197	O	5.41544	-1.80683	-2.53479
H	-7.40444	-1.72869	3.68825	C	4.45546	-2.67797	-0.51528
H	-7.83029	0.66572	4.20511	O	5.34335	-3.72466	-0.17683
H	-9.32506	1.04511	2.38887	C	6.65688	-3.33801	0.21197
H	-6.77875	1.17116	1.39024	C	7.65615	-3.33160	-0.93744
H	-7.10414	3.35115	2.07395	C	8.95192	-2.62309	-0.54605
H	-5.63382	1.86115	4.12492	C	9.87124	-2.42583	-1.74242
H	-4.67248	-0.13672	2.02465	S	11.24427	-1.33165	-1.37281
H	-2.71308	0.60254	2.97018	O	12.02658	-1.24811	-2.62919
H	-3.15307	-0.78560	3.97315	O	10.62357	-0.03722	-0.98897
H	-3.43230	1.94187	4.64879	O	11.98712	-1.97231	-0.26140
H	-5.07564	3.81303	3.92977	H	3.87658	-5.66443	-1.18579
H	-4.35372	5.78038	2.72286	H	2.12358	-7.41294	-1.33081
H	-5.86100	5.51388	0.86377	H	2.08020	-7.53733	-3.70153
H	-3.71428	3.91689	0.39940	H	0.59652	-5.40330	-3.02759
H	-3.09064	5.89292	-0.53924	H	-0.90239	-7.08777	-3.05988
H	-1.87362	5.53129	2.22423	H	-0.10880	-6.37324	-0.23046
H	-2.29368	2.50802	1.96087	H	0.95268	-3.64478	-1.11253
H	-0.68123	4.10424	4.00051	H	0.56647	-4.80752	1.66672
H	0.03587	3.30636	2.58287	H	-0.63262	-3.75567	0.87253
H	-1.43952	1.89530	4.55554	H	-2.05744	-6.85224	-0.87796
H	-0.93413	6.49237	0.58940	H	-4.03314	-6.55225	-2.22810
H	-0.10567	6.62972	-1.64955	H	-2.04573	-5.36612	-3.80690
H	-2.04401	4.59626	-1.96015	H	-3.11294	-3.69532	-2.72756
H	-0.14288	3.61561	-1.89700	H	-5.43810	-3.75530	-3.49475
H	1.14074	4.20187	-3.71526	H	-5.43110	-4.66954	-1.02362
H	2.38235	5.25500	-1.50310	H	-2.76785	-3.36498	-0.25947
H	0.90312	3.49752	0.50193	H	-4.31584	-2.56256	1.25872
H	3.04098	3.14512	1.09431	H	-3.59375	-3.97197	2.08381
H	2.71348	4.62908	1.99969	H	-6.83382	-3.16667	-0.59352
H	4.36358	4.23761	-0.21146	H	-8.05077	-1.46036	-1.93723
H	3.74655	4.12164	-2.75155	H	-7.36014	-2.30360	-4.05621
H	4.07578	2.10476	-4.20305	H	-5.29469	-0.81326	-3.06115
H	1.88885	0.94307	-2.42653	H	-6.66507	0.89825	-4.02959
H	3.27768	-0.82835	-3.96985	H	-7.15165	0.87227	-1.33664
H	4.80617	0.07761	-2.59903	H	-4.49212	-0.46105	-0.63073
H	3.44871	1.48839	-0.27055	H	-4.62239	1.49381	0.64387
H	5.21810	0.16672	0.47323	H	-5.25612	0.21941	1.71882

H	5.81639	1.83515	0.45713	H	-6.79802	2.87687	-1.54650
H	8.56192	0.63643	-1.07431	H	-6.03091	4.56433	-3.17693
H	7.84002	0.62425	0.54581	H	-6.13129	3.14491	-5.09675
H	7.51301	-1.70293	-1.39124	H	-3.80976	2.57822	-3.80615
H	8.95093	-1.58290	-0.39196	H	-3.25709	4.54652	-5.13473
H	7.73731	-1.76013	1.66070	H	-3.64432	5.35071	-2.53620
H	6.17567	-1.52091	0.90417	H	-3.19122	2.58282	-1.30301
H	6.18184	-3.68024	-0.13874	H	-3.49157	5.31561	0.00571
H	7.88599	-3.97694	0.28866	H	-1.92207	4.58231	-0.38434
H	2.55545	2.59083	-6.27122	H	-1.83705	6.22328	-2.76660
H	1.68548	1.20944	-5.55519	H	-0.16851	6.48053	-4.55265
H	0.56160	4.04242	-5.84653	H	-1.41861	5.43042	-6.29592
H	0.17853	2.72012	-6.94266	H	-0.31004	3.44156	-4.71972
H	-1.04367	1.51298	-5.25976	H	1.33505	3.93230	-6.37718
H	-0.26975	2.39590	-3.94133	H	1.96562	5.35086	-4.06914
H	-1.48630	4.45934	-4.55536	H	0.59828	3.40861	-2.14927
H	-2.32330	3.59684	-5.87904	H	2.21673	5.95309	-1.68482
O	9.95052	1.88795	4.05490	H	2.99159	4.36176	-1.89036
O	2.57904	1.13303	2.34812	H	3.78418	4.51719	-4.57020
N	7.90858	0.92153	3.93225	H	4.90339	2.86002	-5.98482
N	-2.43247	0.04457	0.46575	H	3.08462	2.52837	-7.49832
N	-3.33113	-0.89151	0.13939	H	2.59219	1.01199	-5.26911
N	-3.20344	-1.23858	-1.09320	H	4.11483	-0.43183	-6.45314
N	-2.19108	-0.52749	-1.59849	H	5.54786	0.92276	-4.51958
C	8.76019	1.98058	3.79094	H	3.05474	1.46554	-2.83576
C	8.11707	3.24562	3.25602	H	4.57093	0.28131	-1.47089
C	6.68052	3.41707	3.75401	H	4.70420	1.99907	-1.06265
C	5.88696	2.16995	3.46176	H	6.24225	-0.95279	-4.14496
C	4.55200	2.19207	3.08700	H	5.99785	-3.32914	-4.65383
C	3.86126	1.00639	2.80377	H	4.67202	-3.05389	-6.60550
C	4.50756	-0.21938	2.97725	H	2.99905	-2.87725	-4.41571
C	5.84616	-0.24640	3.36588	H	3.25302	-5.30514	-4.50099
C	6.54299	0.93573	3.57838	H	4.87989	-4.43915	-2.59273
C	1.91342	-0.09199	2.03112	H	3.38286	-1.81512	-2.13873
C	0.56708	0.21715	1.40310	H	3.47716	-2.93452	-0.09037
C	0.71515	0.65909	-0.05041	H	4.79106	-1.72496	-0.08116
C	-0.55981	1.20693	-0.70731	H	6.62284	-2.33650	0.66751
C	-1.71256	0.26928	-0.62407	H	6.96981	-4.03962	0.99292
C	-1.78475	-0.65162	-3.01337	H	7.85555	-4.35987	-1.26228
C	-0.40886	-1.31472	-3.13207	H	7.20149	-2.80390	-1.77972
C	-0.03019	-1.53845	-4.60022	H	8.71298	-1.63210	-0.14030
C	-1.12410	-2.28272	-5.36280	H	9.48255	-3.17025	0.24075
C	-2.45244	-1.53479	-5.25848	H	10.29613	-3.36995	-2.09543
C	-2.86423	-1.40056	-3.79437	H	9.33112	-1.96059	-2.57385
H	8.33280	0.04297	4.20334	H	6.63507	-0.41298	-0.89630
H	8.12261	3.16924	2.16068	H	8.06597	0.43209	-1.52271

H	8.75554	4.08442	3.53512
H	6.21387	4.28595	3.28348
H	6.69133	3.59961	4.83622
H	4.03403	3.14052	3.01253
H	4.01053	-1.15929	2.77125
H	6.33011	-1.21317	3.46693
H	1.80384	-0.69094	2.94249
H	2.54110	-0.66452	1.33401
H	0.04709	0.96871	2.00851
H	-0.04288	-0.69221	1.45544
H	1.10854	-0.18175	-0.63558
H	1.46520	1.45396	-0.11560
H	-0.37608	1.45554	-1.75760
H	-0.86930	2.13860	-0.21762
H	-1.74948	0.37465	-3.39260
H	-0.41576	-2.27536	-2.60002
H	0.35447	-0.68747	-2.65598
H	0.91866	-2.08178	-4.64281
H	0.15174	-0.56545	-5.07390
H	-1.24539	-3.28555	-4.92896
H	-0.83329	-2.41542	-6.40945
H	-3.23944	-2.05709	-5.81059
H	-2.35653	-0.53395	-5.69956
H	-3.81143	-0.86436	-3.69574
H	-2.98888	-2.40923	-3.37948

H	7.53960	2.40025	-0.08062
H	6.11772	1.56419	0.55462
H	7.59313	-0.27885	1.42099
H	9.01236	0.61285	0.86241
H	8.26408	2.54521	2.38512
H	6.95199	1.49868	2.96376
H	0.02955	5.06114	0.16605
H	1.05290	4.75658	1.57843
H	1.32501	7.02170	1.79958
H	2.25893	6.98133	0.31755
H	0.52105	8.67507	0.05159
H	0.05326	7.30286	-0.96580
H	-1.41981	6.48892	0.96936
H	-1.06061	8.01057	1.80352
H	-2.28719	3.28264	2.87284
H	-1.38676	4.43053	1.86920
H	-3.40946	6.00585	1.99894
H	-4.06598	4.94341	3.24420
H	-2.09888	5.39020	4.69279
H	-1.24691	6.14559	3.35150
H	-3.05603	7.93057	3.26694
H	-3.67226	7.29575	4.80998
H	-8.05944	0.13517	0.69927
H	-7.50862	0.39308	2.36173
H	-8.53533	2.67525	2.33649
H	-9.04510	2.42902	0.66617
H	-10.46273	0.47901	1.38694
H	-9.97350	0.74188	3.05922
H	-11.03548	3.00695	3.02187
H	-11.51519	2.75251	1.33427
H	-5.25298	-6.10592	0.82745
H	-5.04213	-5.78445	2.55617
H	-7.48770	-5.28201	2.74679
H	-7.68019	-5.56282	1.01618
H	-6.90507	-7.94046	1.32051
H	-6.75290	-7.66541	3.05427
H	-9.20888	-7.20766	3.20370
H	-9.36011	-7.45999	1.45551
H	1.75258	-1.77903	2.92758
H	0.76708	-3.19422	3.31447
H	2.68281	-4.68605	2.64350
H	3.62960	-3.27087	2.21524
H	3.51496	-2.45886	4.59097
H	2.57296	-3.88279	5.03838
H	4.47580	-5.36499	4.42715
H	5.42192	-3.96102	3.90319

O	6.62131	1.10369	8.11485
O	3.87220	0.67244	1.01284
N	5.82121	0.20630	6.19822
N	-1.93580	0.62465	1.85150
N	-2.59154	-0.53405	1.65878
N	-2.44763	-0.94883	0.44876
N	-1.69517	-0.04054	-0.18219
C	6.15281	1.26198	6.99407
C	5.90875	2.62248	6.37303
C	4.65024	2.62937	5.50047
C	4.73977	1.52524	4.47856
C	4.23854	1.62733	3.18090
C	4.35828	0.55717	2.29814
C	5.00568	-0.61224	2.69681
C	5.51696	-0.72254	3.98091
C	5.36185	0.33497	4.87744
C	2.77522	-0.20341	0.73985
C	1.44450	0.40017	1.14253
C	1.01007	1.48477	0.16091
C	-0.39815	2.03084	0.44675
C	-1.37147	0.92877	0.69470
C	-1.26192	-0.25183	-1.56752
C	-0.28566	-1.42738	-1.62864
C	0.24540	-1.60532	-3.05143
C	-0.90968	-1.79355	-4.03787
C	-1.93458	-0.65866	-3.93644
C	-2.44750	-0.47507	-2.50359
H	6.00481	-0.73547	6.54032
H	6.79381	2.83370	5.75977
H	5.85583	3.35448	7.17997
H	4.52596	3.59756	5.00933
H	3.76551	2.47268	6.13176
H	3.74790	2.53741	2.84600
H	5.11978	-1.43183	1.99413
H	6.03793	-1.61477	4.30551
H	2.93924	-1.16239	1.23608
H	2.78998	-0.38404	-0.34364
H	1.51512	0.80152	2.16250
H	0.71465	-0.41834	1.17361
H	1.04911	1.06697	-0.85450
H	1.71305	2.32231	0.17564
H	-0.74388	2.67293	-0.37351
H	-0.38450	2.63645	1.35484
H	-0.75798	0.67617	-1.86027
H	-0.83384	-2.33122	-1.32329
H	0.53177	-1.28931	-0.91045

H	0.92360	-2.46709	-3.08413
H	0.83906	-0.72310	-3.33206
H	-1.40475	-2.74754	-3.81154
H	-0.52957	-1.86836	-5.06096
H	-2.78051	-0.84934	-4.60424
H	-1.47039	0.27896	-4.27149
H	-3.13947	0.37387	-2.44295
H	-2.99769	-1.36787	-2.17442

Table S4. The HOMO and LUMO energies, the corresponding energy gap ($\Delta_{LUMO-HOMO}$), as well as the values of electronic chemical potential (μ), hardness (η) and electrophilicity (ω) presented for the most stable CDs:CIL complexes and their isolated molecules. All values are given in [eV].

Structure	*HOMO*	*LUMO*	$\Delta_{LUMO-HOMO}$	μ	η	ω
**α-CD:CIL_K01**	-7.11	0.17	7.27	-3.47	3.64	1.66
**β-CD:CIL_K01**	-7.24	0.20	7.44	-3.52	3.72	1.66
**HPβ-CD_mol1:CIL_K02**	-7.24	0.20	7.45	-3.52	3.72	1.66
**HPβ-CD_mol2:CIL_K02**	-7.23	0.22	7.44	-3.51	3.72	1.65
**SBEβ-CD_mol1:CIL_K03**	-6.80	0.70	7.50	-3.05	3.75	1.24
**SBEβ-CD_mol2:CIL_K03**	-6.81	0.74	7.55	-3.04	3.77	1.22
**α-CD**	-8.65	2.46	11.11	-3.09	5.56	0.86
**β-CD**	-8.75	2.59	11.33	-3.08	5.67	0.84
**HPβ-CD_mol1**	-8.50	2.14	10.64	-3.18	5.32	0.95
**HPβ-CD_mol2**	-8.64	2.59	11.22	-3.02	5.61	0.81
**SBEβ-CD_mol1**	-8.07	2.61	10.68	-2.73	5.34	0.70

**SBEβ-CD_mol2**	-7.90	2.65	10.54	-2.62	5.27	0.65
**CIL**	-6.90	0.67	7.56	-3.12	3.78	1.28

Table S5. The values of the Gibbs energy of solvation (G_{corr_solv}) obtained for the isolated cyclodextrins.

Molecule	G_{corr_solv}
**α-CD**	-27.73
**β-CD**	-26.82
**HPβ-CD_mol1**	-33.99
**HPβ-CD_mol2**	-34.05
**SBEβ-CD_mol1**	-143.19
**SBEβ-CD_mol2**	-836.47

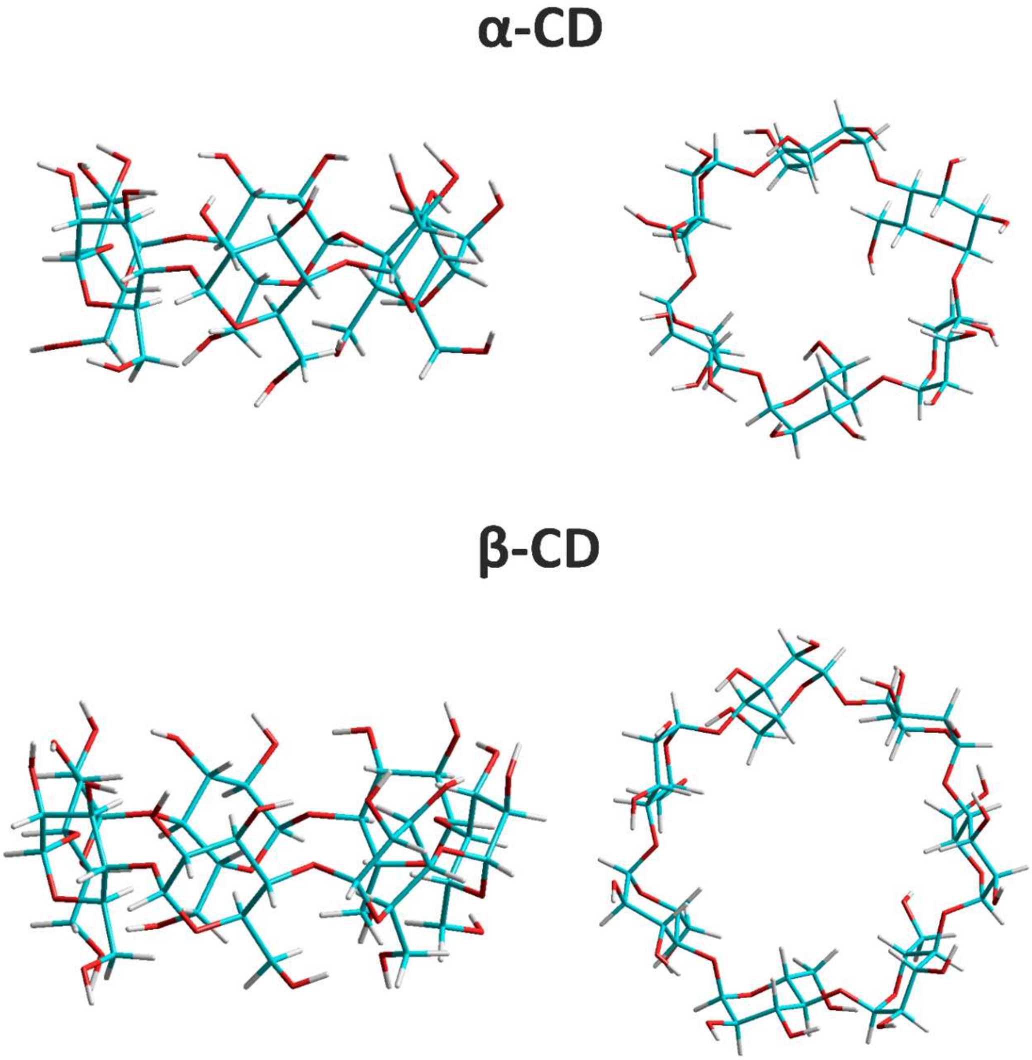

Figure S5. The structures of α-CD and β-CD taken to form the initial models of complexes. The geometry of both cyclodextrins was based on their experimental structures reported in ref. [70,71], for α-CD and β-CD, respectively.

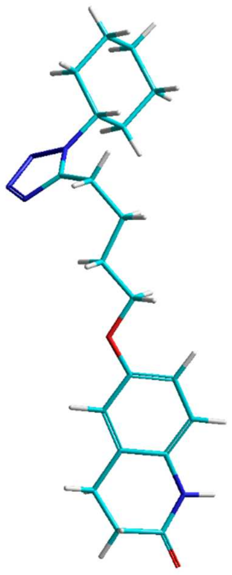

Figure S6. The geometry of cilostazol (**CIL**) taken to form the initial models of complexes. The geometry is based on its experimental geometry reported in ref. [72]

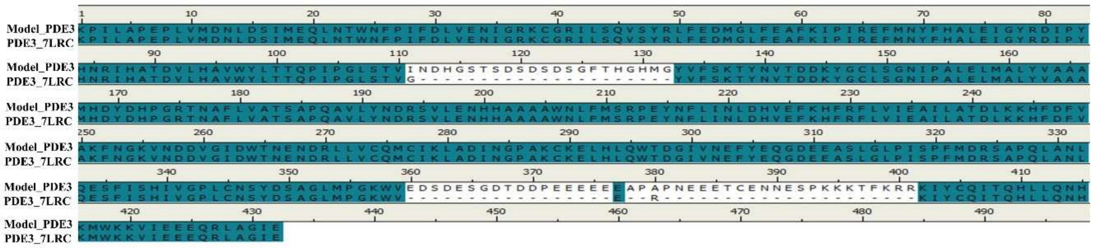

Figure S7. Amino acid sequence comparison between the crystal and the model to show the missing residues.
